# The Spore Differentiation Pathway in the Enteric Pathogen *Clostridium difficile*

**DOI:** 10.1371/journal.pgen.1003782

**Published:** 2013-10-03

**Authors:** Fátima C. Pereira, Laure Saujet, Ana R. Tomé, Mónica Serrano, Marc Monot, Evelyne Couture-Tosi, Isabelle Martin-Verstraete, Bruno Dupuy, Adriano O. Henriques

**Affiliations:** 1 Instituto de Tecnologia Química e Biológica, Universidade Nova de Lisboa, ITQB-UNL, Estação Agronómica Nacional, Oeiras, Portugal; 2 Univ. Paris Diderot, Sorbonne Paris Cité, Cellule Pasteur, Paris, France; 3 Laboratoire Pathogenèse des Bactéries Anaérobies, Institut Pasteur, Paris, France; University of Geneva Medical School, Switzerland

## Abstract

Endosporulation is an ancient bacterial developmental program that culminates with the differentiation of a highly resistant endospore. In the model organism *Bacillus subtilis*, gene expression in the forespore and in the mother cell, the two cells that participate in endospore development, is governed by cell type-specific RNA polymerase sigma subunits. σ^F^ in the forespore, and σ^E^ in the mother cell control early stages of development and are replaced, at later stages, by σ^G^ and σ^K^, respectively. Starting with σ^F^, the activation of the sigma factors is sequential, requires the preceding factor, and involves cell-cell signaling pathways that operate at key morphological stages. Here, we have studied the function and regulation of the sporulation sigma factors in the intestinal pathogen *Clostridium difficile*, an obligate anaerobe in which the endospores are central to the infectious cycle. The morphological characterization of mutants for the sporulation sigma factors, in parallel with use of a fluorescence reporter for single cell analysis of gene expression, unraveled important deviations from the *B. subtilis* paradigm. While the main periods of activity of the sigma factors are conserved, we show that the activity of σ^E^ is partially independent of σ^F^, that σ^G^ activity is not dependent on σ^E^, and that the activity of σ^K^ does not require σ^G^. We also show that σ^K^ is not strictly required for heat resistant spore formation. In all, our results indicate reduced temporal segregation between the activities of the early and late sigma factors, and reduced requirement for the σ^F^-to-σ^E^, σ^E^-to-σ^G^, and σ^G^-to-σ^K^ cell-cell signaling pathways. Nevertheless, our results support the view that the top level of the endosporulation network is conserved in evolution, with the sigma factors acting as the key regulators of the pathway, established some 2.5 billion years ago upon its emergence at the base of the Firmicutes Phylum.

## Author Summary

Endospores, as those formed by the anaerobic Clostridial species, the aerobic *Bacillus* and related organisms, are highly resilient dormant cell types. Endospore development has been extensively studied in *Bacillus subtilis*. The process starts with an asymmetric division that forms a larger mother cell and a smaller forespore. Gene expression in these two cells is primarily governed by four cell type-specific RNA polymerase sigma factors, two active sequentially in the mother cell and two active sequentially in the forespore. The precise timing of sigma factor activation is coordinated between the mother cell and forespore by several mechanisms. By combining studies of gene expression at the single cell level with the phenotypic characterization of mutants for the sigma genes, we describe the regulation of gene expression during endospore development by the intestinal pathogen *C. difficile*. In this organism, the endospore is the vehicle for transmission and persistence in the host and the environment. The overall picture that emerges is of a developmental program in which the coordination of gene expression between the forespore and mother cell is less tight than in *B. subtilis*, in what may be a general feature of endospore formation in the more ancient Clostridia group of bacteria.

## Introduction

Endosporulation is an ancient bacterial cell differentiation program that culminates with the formation of a highly resistant dormant cell, the endospore. Bacterial endospores (hereinafter designated spores for simplicity), as those formed by species of the well-known *Bacillus* and *Clostridium* genera, but also by many other groups within the Firmicutes phylum, resist to extremes of physical and chemical parameters that would rapidly destroy the vegetative cells, and are the most resistant cellular structure known [1], [2]. Their resilience allows them to accumulate in highly diverse environmental settings, often for extremely long periods of time. The range of environments occupied by sporeformers, include niches within metazoan hosts, in particular the gastro-intestinal tract (GIT) (*e.g.* [3]–[5]). *B. subtilis*, for example, a non-pathogenic sporeformer, can go through several cycles of growth, sporulation and germination in the GIT [5]. For pathogenic sporeformers, spores are often the infectious vehicle as in the inhalational or gastric forms of anthrax, the potentially lethal disease caused by *B. anthracis* [6]. Also, it is a protein present at the spore surface that mediates spore internalization by macrophages, and spore dissemination to local lymph nodes, which are central to pathogenesis [6], [7]. Infection by the intestinal human and animal pathogen *C. difficile*, an obligate anaerobe, and the subject of the present investigation, often also starts with the ingestion of spores [8], [9]. *C. difficile* is the causative agent of an intestinal disease whose symptoms can range from mild diarrhea to severe, potentially lethal inflammatory lesions such as pseudomembraneous colitis, toxic megacolon or bowel perforation [8], [10]. Ingested spores of this organism germinate in the colon, to establish a population of vegetative cells that will produce two potent cytotoxins and more spores [8], [10]–[12]. Infection develops because *C. difficile* can colonize the gut if the normal intestinal microbiota is disturbed [8] [9]. Toxinogenesis is responsible for most of the disease symptoms, whereas the spores, which can remain latent in the gut, are both a persistence and transmission factor [8], [10]–[13]. While an asporogeneous mutant of *C. difficile* can cause intestinal disease, it is unable to persist within and transmit between host organism [13]. The spore thus has a central role in persistence of the organism in the environment, infection, recurrence and transmission of the disease. Recent years have seen the emergence of strains, so called hypervirulent, linked to increased incidence of severe disease, higher relapse rates and mortality, and *C. difficile* is now both a main nosocomial pathogen associated with antibiotic therapy as well as a major concern in the community [8], [9], [14].

The basic spore plan is conserved [15]–[17]. The genome is deposited in a central compartment delimited by a lipid bilayer with a layer of peptidoglycan (PG) apposed to its external leaflet. This layer of PG, known as the germ cell wall, will serve as the wall of the outgrowing cell that forms when the spore completes germination. The germ cell wall is encased in a thick layer of a modified form of PG, the cortex, essential for the acquisition and maintenance of heat resistance [15], [16]. The cortex is wrapped by a multiprotein coat, which protects it from the action of PG-breaking enzymes produced by host organisms or predators [15], [16]. In some species, including the pathogens *B. anthracis*, *B. cereus* and *C. difficile*, the coat is further enclosed within a structure known as the exosporium. The coat and the exosporium, when present, mediate the immediate interactions of the spore with the environment, including the interaction with small molecules that trigger germination [7], [15], [18]–[20].

The process of spore differentiation has been extensively studied in the model organism *B. subtilis* [21] [22]. Rod-shaped vegetative cells, growing by binary fission, will switch to an asymmetric (polar) division when facing severe nutritional stress. Polar division yields a larger mother cell and a smaller forespore, the future spore. The mother cell then engulfs the forespore. This process, akin to phagocytosis and a hallmark of endosporulation, isolates the forespore from the surrounding medium, and releases it as a cell, surrounded by a double membrane, within the mother cell cytoplasm [21], [22]. With the exception of the germ cell wall, which is formed from the forespore, the assembly of the main spore protective structures is mostly a function of the mother cell [15], [16]. At the end of the process, and following a period of spore maturation, the mother cell undergoes autolysis, to release the finished spore. For the organisms that have been studied to date, mostly by transmission electron microscopy (TEM), this basic sequence of morphological events appears conserved [15], [21].

The developmental regulatory network of sporulation shows a hierarchical organization and functional logic [17]. A master regulatory protein, Spo0A, activated by phosphorylation, governs entry into sporulation, including the switch to asymmetric division [21], [23]. Gene expression in the forespore and mother cell is controlled by 4 cell type-specific sigma factors, which are sequentially activated, alternating between the two cells. σ^F^ and σ^E^ control the early stages of development in the forespore and the mother cell, respectively, and are replaced by σ^G^ and σ^K^ when engulfment of the forespore is completed [21]–[23]. Activation of the sporulation sigma factors coincides with the completion of key morphological intermediates in the process, at which stages cell-cell signaling events further allow the alignment of the forespore and mother cell programs of gene expression. The result is the coordinated deployment of the forespore and mother cell lines of gene expression, in close register with the course of cellular morphogenesis [21]–[23]. Additional regulatory proteins, working with the sigma factors, generate feed forward loops (FFLs) that create waves of gene expression, minimizing transcriptional noise and impelling morphogenesis forward [17]. A large number of genes of *B. subtilis*, distributed in the four cell type-specific regulons, participate in spore morphogenesis [24]–[27]. The key regulatory factors, Spo0A and the sporulation sigma factors, which define the highest level in the functional and evolutionary hierarchy of the sporulation network, are conserved in sporeformers. The FFLs show an intermediary level of conservation, with the “structural” genes, with the lower level of conservation, at the lowest level in the hierarchy [17], [24]–[27]. The conservation of the sporulation sigma factors has suggested that their role and sequential activation is also maintained across species [17]. However, recent studies have revealed differences in the roles and time of activity of the sigma factors during spore morphogenesis in several Clostridial species [28]–[35]. For instance, σ^F^ and σ^E^ are active prior to asymmetric division in *C. acetobutylicum* and *C. perfringens* [30], [31], [33], [35]. Also, σ^K^, which in *B. subtilis* controls late stages of morphogenesis in the mother cell, is active in pre-divisional cells of C. *perfringens* and *C. botulinum* [29], [34]. Collectively, and relative to the aerobic *Bacilli*, the *Clostridia* represent an older group within the Firmicutes phylum, at the base of which endosporulation has emerged some 2.5 billion years ago, before the initial rise in oxygen levels [24]–[28], [36].

Despite the importance of *C. difficile* for human health and activities, and the central role of sporulation in the infection cycle, a cytological and molecular description of sporulation has been lacking. Here, we have combined cytological and genetics methodologies to define the sequence of sporulation events in *C. difficile* and the function of the cell type-specific sigma factors. In addition, by using a fluorescent reporter for studies of gene expression at the single cell level, we were able to correlate the expression and activity of the sporulation-specific sigma factors with the course of morphogenesis. A key observation is that during *C. difficile* sporulation the forespore and mother cell programs of gene expression are less tightly coupled. Our study also provides a platform for additional studies of the regulatory network and for integrating the expression and function of the effector genes, many of which will be species-specific, and possibly related to host colonization and transmission.

## Results

### Sporulation in sporulation medium (SM)

**Figure 1:**
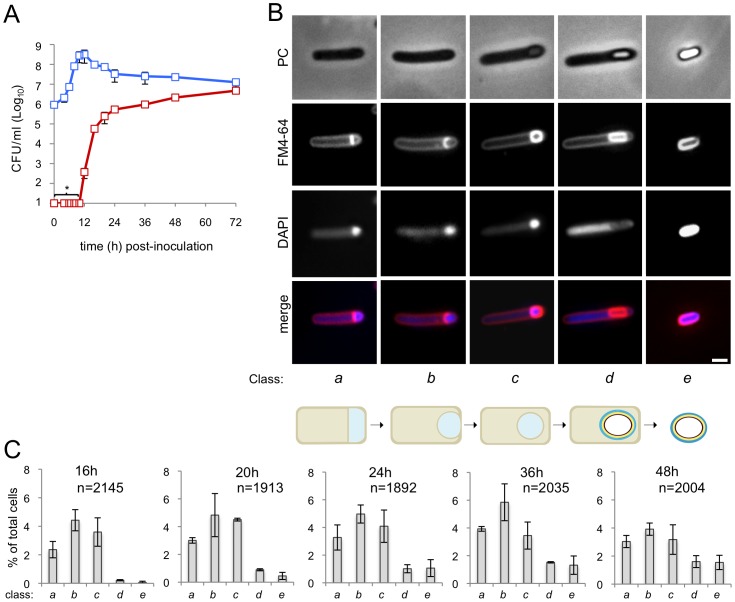
Sporulation in *C. difficile* 630Δ*erm*. (A) The spore (red symbols) and total cell titer (blue symbols) was measured for a culture of strain 630Δ*erm* at the indicated times post-inoculation in SM. The data represent the average ± standard deviation (SD) of three independent experiments. No heat resistant CFUs were detected for an undiluted 100 µl culture sample (CFU/ml: ≤10^1^). (B) Samples of an SM broth culture of strain 630Δ*erm* were collected 24 h after inoculation, stained with DAPI and FM4-64 and examined by phase contrast (PC) and fluorescence microscopy. The panel illustrates the stages in the sporulation pathway, according to the classes defined in the text and represented schematically at the bottom of the panel (see the Results section). Scale bar, 1 µm. (C) Quantification of the percentage of cells in the morphological classes represented in (B) (as defined in the text), relative to the total viable cell population, for strain 630Δ*erm* at the indicated times following inoculation in SM broth. The data represent the average ± SD of three independent experiments. The total number of cells scored (n) is indicated in each panel.

Earlier studies using TEM have suggested that the main stages of sporulation are conserved amongst *Bacillus* and Clostridial species [37]. Here, we examined sporulation of *C. difficile* using phase contrast and fluorescence microscopy with the goal of establishing a platform for both the phenotypic analysis of mutants blocked in the process and for the analysis of developmental gene expression in relation to the course of morphogenesis. This approach requires the individual scoring of a relative large number of cells. However, under culturing conditions widely used for *C. difficile* sporulation, as in BHI medium, supplemented or not with cysteine and yeast extract (BHIS), the process is highly heterogeneous, or asynchronous [11], [14], [38], [39], reviewed by [40]). High titers of spores have been reported following 48 h incubation of liquid cultures in the Sporulation Medium (or SM) described by Wilson and co-authors [41], but how the spore titer developed over time was not reported. More recently, SM was used, with some modifications to the original formulation, for high yield spore production on agar plates [42]. We determined the spore titer during growth of the wild type strain 630Δ*erm* in liquid SM cultures. As shown in Figure 1A, no heat resistant spores could be detected at the time of inoculation, or during the first 10 hours of growth. Heat resistant spores, 3.7×10^2^ spores/ml, were first detected at hour 12, a titer that increased to 2.4×10^5^ at hour 24 (about 14 hours after entry into the stationary phase of growth), the later number corresponding to a percentage of sporulation of 0.3% (Figure 1A and Table S1). From hour 24 onwards, the spore titer increased slowly, to reach 4.7×10^6^ spores/ml 72 hours following inoculation, corresponding to 43.8% sporulation (Figure 1A). Importantly, the percentage of sporulation in SM medium was higher than in BHI or BHIS for all the time points tested (Figure S1). In particular, the titer of spores in SM was two orders of magnitude higher than in BHIS, when measured 24 hours following inoculation (Figure S1). For our studies of spore morphogenesis and cell type-specific gene expression, SM was adopted.

### Stages of sporulation

We then wanted to monitor progress through the morphological stages of sporulation by phase contrast and fluorescence microscopy. In a first experiment, a sample from cultures of the wild type strain 630Δ*erm* was collected 24 hours after inoculation into SM, for microscopic examination following staining with the lipophilic membrane dye FM4-64 and with the DNA marker DAPI. Cells representative of several distinctive morphological classes are shown on Figure 1B (top). Cells with straight asymmetrically positioned septa (class *a*) and cells with curved spore membranes (*i.e.*, at intermediate stages in the engulfment sequence; class *b*), both showing intense staining of the forespore DNA, were readily seen (Figure 1B). Another class comprised cells showing strong uniform FM4-64 staining around the entire contour of the forespore (Figure 1B). The staining pattern suggests that the forespore is entirely surrounded by a double membrane, and therefore that the engulfment sequence was finalized. Those cells in which the forespore shows a continuous, strong FM4-64 signal, but has not yet developed partial or full refractility are considered to have just completed the engulfment process, and define class *c*. A strong, condensed DAPI signal in the forespore was also seen for this class (Figure 1B). Intense, uniform staining of the forespore by FM4-64 was maintained in cells carrying phase grey (partially refractile) or phase bright spores, defining class *d* (Figure 1B). DAPI staining of the forespore DNA was variable for both cells with phase grey or phase bright spores in this class (data not shown). Free spores, at least some of which could be stained with DAPI, define a last morphological class (class *e*) (Figure 1B). The stages of sporulation discerned conform well to the sequence established for *B. subtilis* (Figure 1B, bottom) [15], [21], [37]. Staining of the developing spore by FM4-64 following engulfment completion contrasts with the situation in *B. subtilis*, in which the lipophilic dye does not label engulfed forespores [43] (see also Figure S2A). However, in other organisms, FM4-64 stains the engulfed forespore [3] (see also Text S1). Also, FM4-64 does not stain free spores of *B. subtilis* (which do not have and exosporium) or *B. cereus* (which are surrounded by an exosporium) (Figure S2B), but stains *C. difficile* spores (which also possess an exosporium) (Figure S3). Spore staining by FM4-64 may thus be more related to the composition of the surface layers, rather than to the presence of a specific structure. We note that affinity of FM dyes to the spore coats has been reported [3] (see also Text S1).

Under our culturing conditions, cells belonging to each of the five morphological classes considered (*a* to *e*) were seen at all the time points examined (Figure 1C). This suggests that sporulation is heterogeneous, or asynchronous, in agreement with other results [43], with cells entering the sporulation pathway throughout the duration of the experiment. Surprisingly, the representation of cells at intermediate stages in development (classes *a* to *c*) decreased from hour 36 to hour 48, without a corresponding rise in later morphological classes (class *d*, phase grey/bright spores and class *e*, free spores) (Figure 1C). However, Live/Dead staining evidenced cell lysis, including of cells at intermediate stages of sporulation (classes *a* to *c*), from hour 36 of growth onwards (data not shown). As assessed by Live/Dead staining and fluorescence microscopy, lysis of sporulating cells was only marginal at hour 24 of growth (data not shown). Therefore, in subsequent experiments, sporulating cells were scored 24 hours following inoculation. At hour 24, the total number of sporulating cells (*i.e.*, the sum of classes *a* to *e* in Figure 1C) represents about 15% of the total cell population.

### Disruption of the genes for the sporulation sigma factors

**Figure 2:**
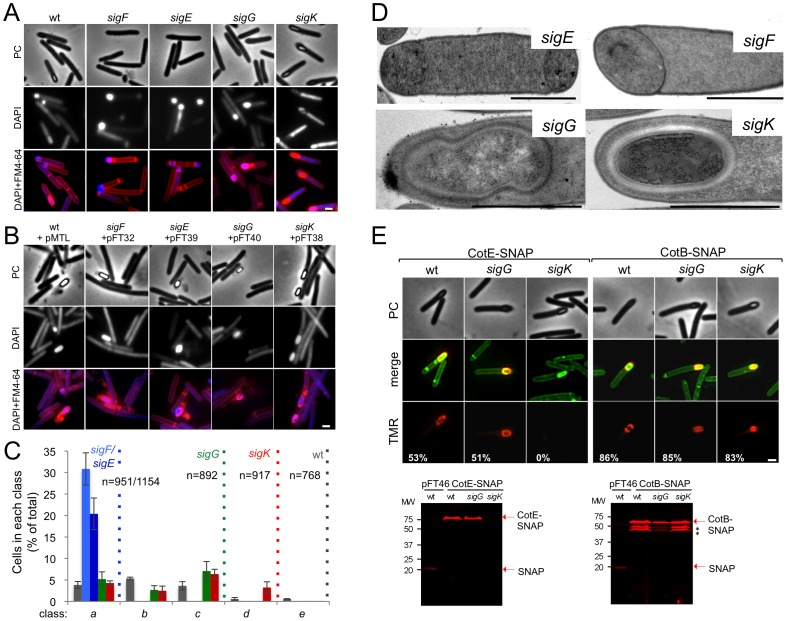
The sporulation pathway in *C. difficile* 630Δ*erm*, and the role of *sigF*, *sigE*, *sigG* and *sigK*. Phase contrast (PC) and fluorescence microscopy analysis of spore morphogenesis in the following strains: (A) 630Δ*erm* (wild type, wt) and the congenic *sigF*, *sigE*, *sigG* and *sigK* mutants; (B) the *sig* mutants bearing the indicated plasmids or the wt strain carrying the empty vector pMTL84121. Cells were collected 24 (A) or 48 h (B) after inoculation in SM broth, and stained with DAPI and FM4-64, prior to microscopic examination. (C) Quantification of the cells in each morphological class, as defined in Figure 1, in the experiment documented in panel A, for the wt strain and the *sig* mutants. The data represent the average ± SD of three independent experiments. The total number of cells analysed (n) is indicated for each strain. (D) TEM images of *sigF*, *sigE*, *sigG* and *sigK* mutant cells. The Images are representative of the most common morphological phenotype observed for each mutant. (E) Fluorescence microscopy of 630Δ*erm* (wt) and *sigG* and *sigK* strains carrying CotE- and CotB-SNAP fusions. Cells were collected 24 h after inoculation in SM medium and labeled with the SNAP substrate TMR-Star (red channel) and the membrane dye MTG (green channel), with which a membrane-staining pattern similar to FM4-64 was obtained. The numbers on the bottom panel represent the percentage of cells which have completed the engulfment process that show localization of the protein fusions around the forespore. Data shown are from one representative experiment in which 80–100 cells were analysed for each strain. Scale bar in panels (A, B, D, E), 1 µm. Total cell extracts were prepared from 24 h SM cultures of derivatives of the 630Δ*erm*, *sigG* and *sigK* strains producing the CotE- (left) and CotB-SNAP (right) fusions, immediately after labeling with TMR-Star. Proteins (30 µg) were resolved by SDS-PAGE and the gel scanned using a fluorimager. Production of the SNAP protein in the background of 630Δ*erm* strain from the P*_tet_* promoter (P*_tet_*-*SNAP^Cd^*, in pFT46; see Text S1), was used as a control. The position of the SNAP or SNAP fusion proteins is indicated by arrowheads. Asteriks indicate possible degradation products.

The genes for the four cell type-specific RNA polymerase sigma factors known to control gene expression during spore differentiation in *B. subtilis* are conserved in sporeformers [24]–[27]. Moreover, their operon structure and genomic context is also maintained (Figure S4A). To investigate whether the function of the σ^F^, σ^E^, σ^G^ and σ^K^ factors is conserved, each of the corresponding genes was disrupted (Figure S4). For this purpose, type II introns were targeted to each of the *sig* genes, using the ClosTron system [44]. As shown in Figure S4A, re-targeting of the intron resulted in insertion after codon 153 of the *sigF* gene, codon 151 of *sigE*, codon 182 of *sigG*, and codon 34 of the 5′-end of the split *sigK* gene, interrupted by the *skin^Cd^* element. Correct insertion of the intron was verified, in all cases, by PCR, and Southern blot analysis showed the presence of a single intron insertion in the genome of the different mutants (Figure S4B through E). The *sig* mutants, along with the parental 630Δ*erm* strain, were induced to sporulate in SM, and the titer of heat resistant spores assessed after 24, 48, and 72 hours of growth. For the wild type strain 630Δ*erm*, the titer of spores was of 3×10^5^ spores/ml at hour 24, 2×10^6^ spores/ml at hour 48, and of 1×10^7^ spores/ml at hour 72 (Table 1). In contrast, no heat resistant spores were found, at any time point tested, for the *sigF* (AHCD533), *sigE* (AHCD532), or *sigG* (AHCD534) mutants. However, a titer of 10^3^ heat resistant spores/ml of culture was found for the *sigK* mutant AHCD535 at hour 72 (Table 1). For complementation studies, we generated multicopy alleles of the *sig* genes, based on replicative plasmid pMTL84121 [45], expressed from their native promoters (the extent of the promoter fragments is shown in Figure S4A). Note that for complementation of the *sigK* mutation the two halves of the gene, together with a short *skin^Cd^* element composed only of the putative recombinase gene (*spoIVCA*, or CD12310) was used (see below for a more detailed description on the complementation of the *sigK* mutation). When measured at hour 72 of growth in SM, the heat resistant spore titer was of 1.7×10^6^ spores/ml for the wild type strain 630Δ*erm* carrying the empty vector pMTL84121. Derivatives of pMTL84121 carrying the *sigF*, *sigE*, *sigG* or *sigK* genes (the later plasmid, pFT38, with the short *skin^Cd^* allele) restored spore formation to the *sig* mutants, as assessed by microscopy (Figure 2B). The same plasmids largely restored heat resistant spore formation to the *sig* mutants (1.6×10^4^, 8.3×10^5^, 3.9×10^5^, 4.8×10^5^ spores/ml for the *sigF*, *sigE*, *sigG*, and *sigK* mutants, respectively, also measured at hour 72 of growth in SM).

**Table 1:** Total and heat resistant (Heat^R^) cell counts (CFU/ml) for the wild type strain (630Δ*erm*) and congenic *sigF*, *sigE*, *sigG* and *sigK* mutants in SM.

Time (h)	630Δ*erm*		*sigF*		*sigE*		*sigG*		*sigK*	
	Total	Heat^R^	Total	Heat^R^	Total	Heat^R^	Total	Heat^R^	Total	Heat^R^
24	7.3×10^7^±1.6×10^7^	3.1×10^5^±5.6×10^4^	2.3×10^8^±6.4×10^7^	0	9.6×10^7^±7.2×10^7^	0	9.0×10^7^±4.5×10^7^	0	4.4×10^7^±2.7×10^7^	0
48	4.2×10^7^±8.5×10^6^	2.4×10^6^±3.1×10^5^	7.9×10^7^±6.3×10^7^	0	1.3×10^7^±7.8×10^7^	0	9.6×10^6^±4.5×10^6^	0	1.2×10^7^±2.9×10^6^	2.1×10^2^±3.6×10^1^
72	2.2.×10^7^±2.9×10^6^	1.5×10^7^±6.8×10^6^	4.3×10^7^±2.1×10^7^	0	7.1×10^6^±2.8×10^6^	0	3.0×10^6^±3.9×10^6^	0	6.1×10^6^±4.3×10^7^	1.7×10^3^±6.0×10^2^

Note: values represent the average±SD of three independent experiments.

### Morphological characterization of the *sigF*, *sigE*, and *sigG* mutants

To establish the morphological phenotype of the various mutants we used phase contrast and fluorescence microscopy of samples collected from SM cultures at hour 24, labeled with DAPI and FM4-64 (Figure 2). These studies revealed that the *sigF* and *sigE* mutants were blocked at the asymmetric division stage (Figure 2A and C). As previously found for *B. subtilis* [37], [46], [47] both mutants formed abortive disporic forms, and occasionally multiple closely located polar septa (Figure 2A). In addition, for the *sigF* mutant, small round cells were found, probably resulting from detachment of the forespore (Figure 2A). In both mutants, the DNA stained strongly in the forespore(s) and gave a diffuse signal throughout the mother cell (Figure 2A). TEM analysis confirmed the block at the asymmetric division stage for the two mutants (Figure 2D).

Cells of the *sigG* mutant completed the engulfment sequence, but did not proceed further in morphogenesis (Figure 2A and C). As for class *c* in the wild type (Figure 1B, and text above), the forespores in the *sigG* mutant stained strongly with FM4-64 (Figure 2A). TEM of sporulating cells of the *sigG* mutant confirmed engulfment completion, but also revealed deposition of electrodense material around the forespore protoplast (Figure 2D). This deposit could represent coat material. By comparison, no accumulation of electrodense coat-like material is seen by TEM around the engulfed forespore of a *B. subtilis sigG* mutant [48]. In this organism, coat assembly as discernible by TEM, is a late event that requires activation of σ^K^ in the mother cell [15], [16], [37]. Importantly, activation of σ^K^ is triggered by σ^G^, and coincides with engulfment completion [49], [50]. Therefore, the possible accumulation of coat material in the *sigG* mutant could imply that in *C. difficile*, σ^K^ is active independently of σ^G^. We therefore wanted to test whether coat material was deposited around the forespore in the *C. difficile sigG* mutant. In *B. subtilis*, studies of protein localization have relied mainly on the use of translational fusions to the *gfp* gene, or its variants (e.g., [51]). However, an obstacle to the use of *gfp* or its derivatives in the anaerobe *C. difficile*, is that formation of the GFP fluorophore involves an oxidation reaction [52]. For this reason, we turned to the SNAP-tag reporter, which reacts with fluorescent derivatives of benzyl purine or pyrimidine substrates, and has been used in anaerobic bacteria [53], [54]. We designed a variant of the *SNAP26b* gene, termed *SNAP^Cd^*, codon-usage optimized for expression in *C. difficile* (see Materials and Methods; see also Text S1), and used it to construct C-terminal fusions of the SNAP-tag to spore coat proteins CotE and CotB [55], [56] in plasmid pFT58 (Figure S5B and C). The fusions were introduced, in a replicative plasmid, in strain 630Δ*erm* and the *sigG* and *sigK* mutants, and samples from SM cultures at hour 24 were labeled with the cell-permeable fluorescent substrate TMR-Star (see Materials and Methods). Using fluorescence microscopy and fluorimaging of SDS-PAGE-resolved whole cell extracts, no accumulation of CotE-SNAP was detected in cells of a *sigK* mutant, suggesting that the *cotE* gene is under the control of σ^K^ (Figure 2E; see also below). CotB-SNAP, however, accumulated in cells of a *sigK* mutant (Figure 2E), but not in cells of a *sigE* mutant (data not shown), suggesting that expression of *cotB* is under the control of σ^E^. Both CotE-SNAP and CotB-SNAP localized around the forespore in both wild type and in *sigG* cells (Figure 2E). SDS-PAGE and fluorimaging suggested instability of CotB-SNAP for which several possible proteolytic fragments were detected, all of which larger that the SNAP domain (Figure 2E). That no release of a labeled SNAP domain was detected for either protein implies that the localized fluorescence signal is largely due to the fusion proteins. Thus, both early (CotB) and late (CotE) coat proteins are assembled around the forespore in cells of a *sigG* mutant. In all, the results suggest that σ^K^ is active independently of σ^G^, and thus, that the later regulatory protein is not a strict requirement for deposition of at least some coat in *C. difficile*.

### Functional analysis of the *sigK* gene

**Figure 3:**
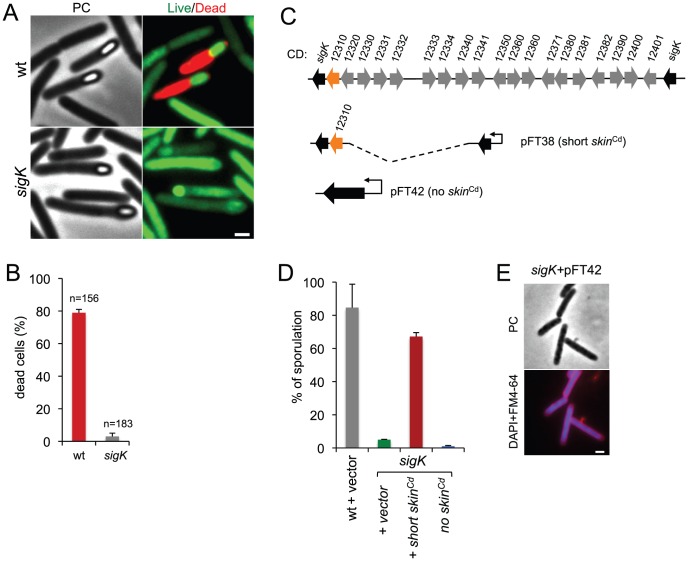
Functional analysis of the *sigK* gene. (A) Live/dead assay for the wild type (630Δ*erm*) and the *sigK* mutant. Shown are phase contrast and the merge between syto 9- (live cells stain; green) and propidium iodide- (dead cells stain; red) stained cells collected at 24 h of growth in SM broth. In the wild type, but not in the *sigK* mutant, development of spore refractility is accompanied by loss of mother cell viability. (B) Percentage of the sporulating cells of the wild type and *sigK* mutant strains (i.e., with visible spores) showing signs of mother cell lysis (red; propidium iodide staining) as scored by direct microscopic observation, 24 hours after inoculation in SM medium. Values are the average ± SD of three independent experiments; “n” represents the total number of cells analyzed. (C) The *sigK-skin* region of the 630Δ*erm* chromosome and plasmids used to complement *sigK* mutant strain. Replicative plasmid pFT38 carries *sigK* interrupted by a shorter version of the *skin* ^Cd^ element, which includes the gene (CD12310) for the recombinase (in orange). Replicative plasmid pFT42 carries an uninterrupted *sigK* gene. The coding sequences are numbered according to the reanotation of the *C.difficile* genome [91]. (D) Percentage of sporulation for strains 630Δ*erm* (wt), *sigK* and *sigK* bearing either pFT38 or pFT42. The indicated percentages are the ratio between the titer of heat resistant spores and the total cell titer, measured 72 h following inoculation in SM medium. Values are the average ± SD of three independent experiments. (E) Fluorescence microscopy showing the phenotype of *sigK* bearing pFT42. Cells were collected at 72 h of growth in SM broth, stained with DAPI and FM4-64, and viewed by phase contrast (PC) and fluorescence microscopy. Scale bar in (A) and (E), 1 µm.

Phase contrast microscopy revealed the presence of some phase bright or partially phase bright spores in SM cultures of the *sigK* mutant, although free spores were only rarely seen (Figure 2A). The ellipsoidal spores were often positioned slightly tilted relative to the longitudinal axis of the mother cell (Figure 2 and 3). The appearance of phase bright spores normally correlates with synthesis of the spore cortex PG, and the development of spore heat resistance [37], [57], in line with the finding that the *sigK* mutant formed heat resistant spores (above). TEM revealed the presence of a cortex layer in cells of the *sigK* mutant, supporting the inferences drawn on the basis of the phase contrast microscopy and heat resistance assays (Figure 2A to E). The number of phase bright or phase grey spores by phase contrast microscopy, was 3.2% of the total number of cells scored at hour 24 of growth in liquid SM (Figure 2C). This is higher than the percentage of sporulation, 0.03%, measured by heat resistance (Table 1). Because full heat resistance requires synthesis of most of the cortex structure, this observation suggests that a large number of the spores formed have an incomplete or dysfunctional cortex. However, we cannot discard the possibility that spores of the mutant are deficient in germination. In any event, unlike in *B. subtilis*, where a *sigK* mutant is unable to form the spore cortex [37], [57], σ^K^ is not obligatory for the biogenesis of this structure in *C. difficile*. In contrast, the TEM analysis did not reveal deposition of coat material around the cortex in cells of the *sigK* mutant (Figure 2D). Although coat assembly most likely starts early, under the control of σ^E^ ([15], [16], [42]; above) the TEM data, together with the data on assembly of CotE (Figure 2E), suggest that the late stages in the assembly of the coats are under σ^K^ control. That free spores were only rarely seen for the *sigK* mutant, prompted us to test whether σ^K^ could have a role in mother cell lysis, using a Live/Dead stain and fluorescence microscopy. In the wild type strain 630Δ*erm*, development of refractility coincided with loss of viability of the mother cell (strong staining with propidium iodide) and strong staining of the developing spore with the Syto 9 dye (Figure 3A), [58]. In contrast, the mother cell remained viable in the *sigK* mutant (strong staining with Syto 9) (Figure 3A and B), and the spores stained only weakly with the Syto 9 dye.

Lastly, our complementation analysis of the *sigK* mutant provided additional functional insight. While wild type levels of sporulation could be restored to a *sigK* mutant by a copy of the *sigK* gene bearing a deletion of all the genes within the *skin^Cd^* element but the recombinase gene (Figure 3C; see above), an uninterrupted copy of the gene, in plasmid pFT42, did not restore sporulation (Figure 3C and D). An earlier study has suggested that the absence of *skin^Cd^* correlates with a sporulation defect and that a *skin^Cd^* ^-^ allele of *sigK* is dominant over the wild type [39]. Our results support the view that generation of an intact *sigK* gene through SpoIVCA-mediated excision of the *skin^Cd^* element is essential for sporulation. Moreover, we found that introduction of the multicopy *skin*-less allele in strain 630Δ*erm* blocked sporulation at an early stage, as no asymmetrically positioned septa could be seen in the transformed strain (Figure 3E). The results suggest that the absence of *skin^Cd^* allows the production of active σ^K^ in pre-divisional cells, and that active σ^K^ interferes with the events leading to asymmetric septation in *C. difficile*.

### Localizing the expression of the sporulation-specific *sig* genes

Having established the main features of sporulation under our culturing conditions, as well as the phenotypes associated with disruption of the *sig* genes, we next wanted to examine cell type-specific gene expression in relation to the course of morphogenesis. As a first step, we examined the expression of the genes coding for σ^F^, σ^E^, σ^G^, and σ^K^ using the *SNAP^Cd^* cassette as a transcriptional reporter. In control experiments, detailed in Text S1, in which expression of *SNAP^Cd^* was placed under the control of the anhydrotetracycline-inducible promoter P*_tet_* (Figure S5A) [59], we showed that complete labeling of all the SNAP produced could be achieved; furthermore, no background was detected for non-induced but labeled cells, or for unlabeled cells producing the SNAP reporter, by either fluorescence microscopy or the combination of fluorimaging and immunobloting with an anti-SNAP antibody, of SDS-PAGE resolved whole cell extracts (Figure S6).

**Figure 4:**
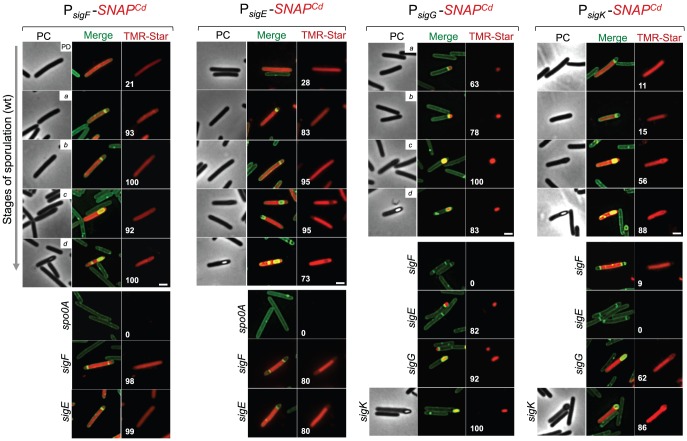
Temporal and cell type-specific expression of *sigF*, *sigE*, *sigG* and *sigK* during sporulation. Microscopy analysis of *C. difficile* cells carrying fusions of the *sigF*, *sigE*, *sigG* and *sigK* promotors to *SNAP^Cd^* in strain 630Δ*erm* (wt) and in the indicated mutants. The cells were collected after 24 h of growth in SM liquid medium, stained with TMR-Star and the membrane dye MTG, and examined by phase contrast (PC) and fluorescence microscopy to monitor SNAP production. The merged images shows the overlap between the TMR-Star (red) and MTG (green) channels. The panels are representative of the expression patterns observed for different stages of sporulation, ordered from early to late for the wild type strains according to the morphological classes *a*-*d* defined in Figure 1, as indicated. For the *sig* strains, the morphological stage characteristic of each mutant is indicated. An extra class that accounts for pre-divisional cells (PD) was introduced for the analysis of both *sigF* and *sigE* transcription. The numbers refer to the percentage of cells at the represented stage showing SNAP fluorescence. The data shown are from one representative experiment, of three performed independently. The number of cells analysed for each class, n, is as follows: PD, n = 100–150; class *a*, n = 30–50; class *b*, n = 50–60; class *c*, n = 30–40; class *d*, n = 15–25; for *sigF/E* mutants, n = 80–120; for *sigG* and *sigK* mutants, n = 40–50. Scale bar: 1 µm.

**Figure 5:**
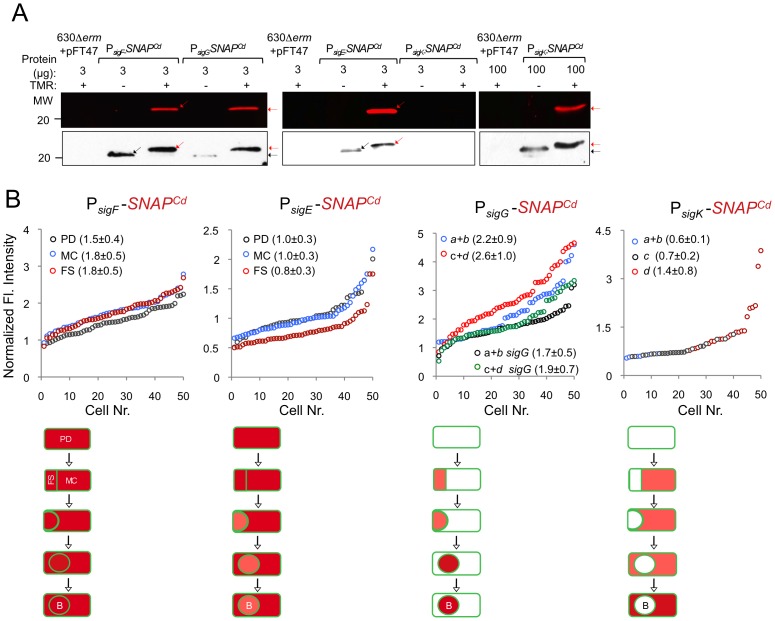
Quantitative analysis of *sigF*, *sigE*, *sigG* and *sigK* expression during sporulation. (A) Whole cell extracts were prepared from derivatives of strain 630Δ*erm* bearing the indicated plasmids or fusions, immediately after labeling with TMR-Star, indicated by the “+” sign (the “−” sign indicates control, unlabeled samples). The indicated amount of total protein was resolved by SDS-PAGE, and the gels scanned on a fluorimager (top) or subject to immunoblotting with anti-SNAP antibodies (bottom). Black and red arrows point to unlabeled or TMR-Star-labeled, respectively, SNAP. Strain 630Δ*erm* carrying pFT47 (empty vector) was used as a negative control for SNAP production. The position of molecular weight markers (in kDa) is indicated. (B) Quantitative analysis of the fluorescence (Fl.) intensity in different cell types of the reporter strains for *sigF*, *sigE*, *sigG* and *sigK* transcription, as indicated. The numbers in the legend represent the average ± SD of fluorescence intensity for the cell class considered (n = 50 cells analysed for each morphological cell class). The data shown are from one experiment, representative of three independent experiments. Schematic representation of the deduced spatial and temporal pattern of transcription (with darker red denoting increased transcription) is shown for each transcriptional fusion. The cell membrane is represented in green. PD, pre-divisional cell; MC, mother cell; FS, forespore; B, phase bright spore; *a* to *d*: sporulation classes as defined in Figure 1.

The promoter regions of *sigF*, *sigE, sigG*, and *sigK* genes were cloned in the *SNAP^Cd^*-containing promoter probe vector pFT47 (Figure S5B). The upstream boundaries of the promoter fragments fused to *SNAP^Cd^* coincide with the 5′-end of the fragments used for the successful complementation of the various *sig* mutants (Figure 3C and S4A; see above). To monitor the production of SNAP during *C. difficile* sporulation, samples of cultures expressing each of the promoter fusions were collected at 24 h of growth in SM medium, and the cells doubly labeled with TMR-Star and the membrane dye MTG, to allow identification of the different stages of sporulation. These were defined based on Figure 1B, with the addition of a class of pre-divisional cells (no signs of asymmetric division). Expression of the various P*_sig_*-*SNAP^Cd^* transcriptional fusions could thus be correlated to the stage in spore morphogenesis. Expression of both *sigF* and *sigE* was first detected in pre-divisional cells of the wild type strain 630Δ*erm*, but not in cells of a *spo0A* mutant (Figure 4), consistent with previous reports [60]–[62] (Figure S7A and B). Both genes continued to be expressed following asymmetric division, in the forespore and the mother cell of both the wild type, and the *sigF* or *sigE* mutants (Figure 4). In these experiments, complete labeling of the SNAP protein was achieved, as revealed by fluorimaging and immunobloting of SDS-PAGE resolved proteins in whole cell extracts (Figure 5A). Quantification of the fluorescence signal shows that while for *sigF* the average intensity did not differ much between forespores (1.8±0.5), and mother cells (1.8±0.5), it increased in both the forespore and the mother cell relative to pre-divisional cells (average signal, 1.5±0.4) (p<0.01). Transcription of *sigE*, in turn, was lower in the forespore (average signal, 0.8±0.3) as compared to pre-divisional cells (1.0±0.3) or the mother cell (1.0±0.3) (p<0.0001) (Figure 5B). Thus, transcription of *sigE*, seems to occur preferentially in the mother cell. Transcription of both *sigF* and *sigE* persisted in both the forespore and the mother cell until a late stage of sporulation, when the forespore becomes phase bright (Figure 4).

In contrast to *sigF* and *sigE*, transcription of *sigG* and *sigK* was confined to the forespore and to the mother cell, respectively (Figure 4). Transcription of *sigG* is detected in the forespore just after asymmetric division, consistent with the presence of a σ^F^-type promoter in its regulatory region (Figure S7C). In agreement with this inference, expression of P*_sigG_*-*SNAP^Cd^* was not detected in cells of a *sigF* mutant (Figure 4). Transcription of *sigG* was detected until the development of spore refractility (Figure 4). Fluorimaging and immunoblot analysis of whole cell extracts shows that under the conditions used, all of the SNAP protein detected was labeled (Figure 5A). In *B. subtilis*, σ^F^ initiates transcription of *sigG* in the forespore [48], [63]. However, transcription of *sigG* also depends on σ^E^, by an unknown mechanism [64]. In contrast, forespore-specific expression of P*_sigG_*-*SNAP^Cd^* was detected in most cells (82%) of a *sigE* mutant (Figure 4). In *B. subtilis*, the main period of *sigG* transcription takes place following engulfment completion, and relies on a positive auto-regulatory loop [65]. We detected transcription of *sigG* both prior and following engulfment completion in a *sigG* mutant (Figure 4). However, the quantitative analysis of the SNAP-TMR signal shows an increase in the average fluorescence intensity following engulfment completion (classes *c*+*d*, 2.6±1.0 as opposed to 2.2±0.9 for classes *a*+*b*) (p<0.01) (Figure 5B). Moreover, the average fluorescence signal for engulfed forespores of a *sigG* mutant suffered a higher reduction compared to the wild type (classes *c+d*, 1.9±0.7 for the mutant as compared to 2.6±1.0 for the wild type; p<0.01), than did the signal for pre-engulfment forespores of the mutant (classes *a+b*, 1.7±0.5 as opposed to 2.2±0.9; p<0.05) (Figure 5B). While evidencing that σ^G^ contributes to transcription of its own gene both prior to and following engulfment completion, these results suggest that the auto-regulatory effect is stronger at the later stage.

In *C. difficile*, transcription of *sigK* was confined to the mother cell and detected soon after asymmetric division (Figure 4). Moreover, disruption of *sigE* resulted in undetected expression of P*_sigK_*-*SNAP^Cd^* (Figure 4). Together, the results suggest that the initial transcription of *sigK* is activated by σ^E^ in the mother cell, consistent with the presence of a possible σ^E^-recognized promoter in the *sigK* regulatory region (Figure S7D). Interestingly, transcription of the *sigK* gene was also detected in a small percentage (9%) of the sporulating cells of a *sigF* mutant (Figure 4). This was unexpected because in *B. subtilis*, activation of σ^E^ in the mother cell is dependent on σ^F^ [66], [67]. This observation thus raises the possibility that the activation of σ^E^ in *C. difficile* is at least partially independent of σ^F^ (see also the following section). Transcription of *sigK* was also detected following engulfment completion, in cells carrying phase grey and phase bright spores (Figure 4). As shown in Figure 5A, all of the SNAP produced from the P*_sigK_*-*SNAP^Cd^* fusion was, under our experimental conditions, labeled. The average intensity of the fluorescence signal from P*_sigK_*-*SNAP^Cd^* in cells prior (classes *a*+*b*, 0.6±0.1) and after engulfment completion (class *c*, 0.7±0.2) was very close. However, expression was significantly increased for those cells that carried phase bright spores (class *d*, 1.4±0.8; p<0.001) (Figure 5B). This suggests that the onset of the main period of *sigK* transcription coincides with the final stages in spore morphogenesis. Lastly, under our experimental conditions, we found no evidence for auto-regulation of *sigK* transcription, as expression of P*_sigK_*-*SNAP^Cd^* was not curtailed by mutation of *sigK* at any morphological stage analyzed (Figure 4 and data not shown).

### Localizing the activity of σ^F^ and σ^E^

**Figure 6:**
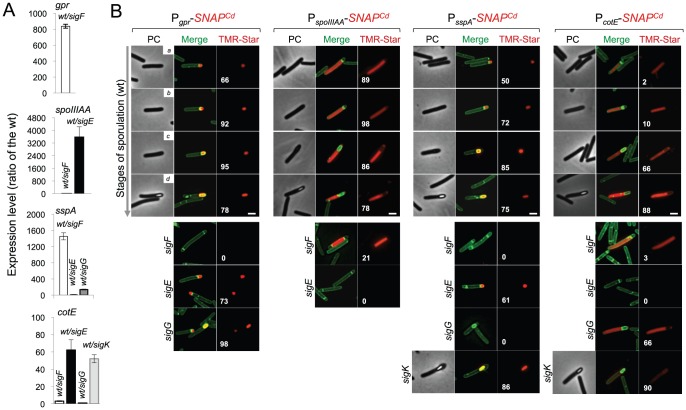
Dependencies for the activation of the cell type-specific sporulation sigma factors. (A) qRT-PCR analysis of *gpr*, *spoIIIAA*, *sspA* and *cotE* gene transcription in strain 630Δ*erm* (wt), and in congenic *sigF*, *sigE*, *sigG* and *sigK* mutants. RNA was extracted from cells collected 14 hours (*gpr* and *spoIIIAA*), 19 hours (*sspA*) and 24 hours (*cotE*) after inoculation in SM liquid medium. Expression is represented as the fold ratio between the wild type (wt) and the indicated mutants. Values are the average ± SD of two independent experiments. (B) Cell type-specific expression of transcriptional fusions of the *gpr*, *spoIIIAA*, *sspA* and *cotE* promoters to *SNAP^Cd^* in the wild type and in the indicated congenic mutants. For each of the strains, expressing the indicated fusions, cells were collected from SM cultures 24 h after inocculation and labeled with TMR-Star (red) and with the membrane dye MTG (green). Following labeling, the cells were observed by phase contrast (PC) and fluorescence microscopy. Merge is the overlap between the TMR-Star (red) and MTG (green) channels. The images are ordered, and the morphological classes defined as in the legend for Figure 4. The numbers refer to the percentage of cells at the represented stage showing SNAP fluorescence. The data shown are from one experiment, representative of three independent experiments. The number of cells analysed for each class, n, is as follows: class *a*, 30–50; class *b*, n = 50–60; class *c*, n = 30–40; class *d*, n = 15–25; for *sigF/E* mutants, n = 80–120; for *sigG* and *sigK* mutants, n = 40–50. Scale bar: 1 µm.

**Figure 7:**
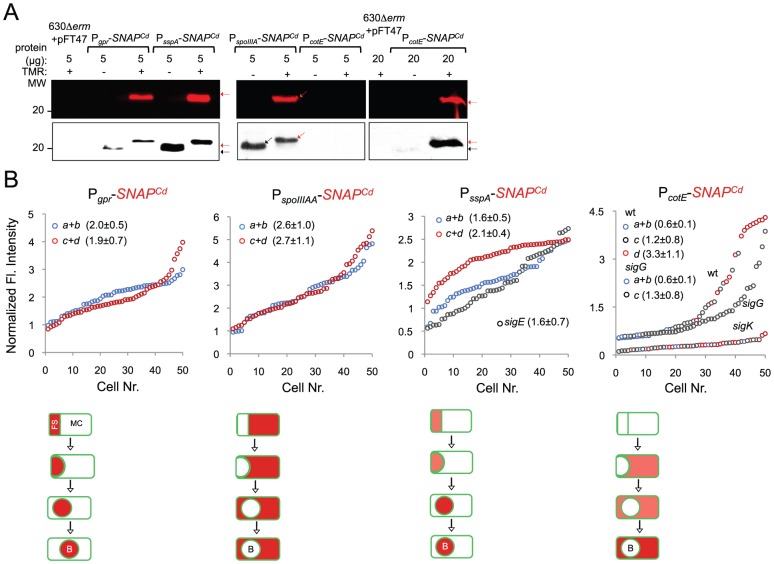
Quantitative analysis of *sigF*, *sigE*, *sigG* and *sigK* activities during sporulation. (A) SDS-PAGE gel and Western Blot analysis of extracts from 630Δ*erm* carrying fusions of *gpr*, *spoIIIAA*, *sspA* and *cotE* to SNAP. Black arrows point to unlabeled SNAP^Cd^ protein, while red arrows point to SNAP after TMR-Star labeling (distinguishable in the WB from the unlabeled form by a shift in protein migration). The TMR-Star fluorescent signal from the SDS-PAGE gel was obtained using a fluorimager. TMR-Star incorporation as well as the amount of protein loaded is indicated for each lane. 630Δ*erm* carrying pFT47 empty vector was used as a negative control of SNAP production. (B) Quantitative analysis of the SNAP fluorescence (Fl.) signal in different cell types of the reporter strains for σ^F^, σ^E^, σ^G^ and σ^K^ activity, as indicated. The numbers in the legend represent the average ± SD of fluorescence intensity for the cell class considered (NB: 50 cells were analysed for each cell type). The average fluorescence intensity (all classes included) from P*_cotE_*-*SNAP^Cd^* is 1.9±1.3 for the wild type, 1.3±0.8 for a *sigG* mutant and 0.3±0.1 for the *sigK* mutant. Data shown are from one experiment, representative of three independent experiments. Schematic representation of the deduced spatial and temporal pattern of transcription is shown for the different fusions (with darker red denoting increased transcription). The cell membrane is represented in green. No activity was seen in predivisional cells for any of the σ factors (not represented). PD, pre-divisional cell; MC, mother cell; FS, forespore; B, phase bright spore; *a* to *d*: sporulation classes ordered and defined as in the legend for Figure 4.

To investigate the genetic dependencies for sigma factor activity during sporulation in *C. difficile*, we used transcriptional *SNAP^Cd^* fusions to promoters under the control of each cell type-specific sigma factor. These promoters were selected on the basis of qRT-PCR experiments and the presence on their regulatory regions, of sequences conforming well to the consensus for promoter recognition by the sporulation sigma factors of *B. subtilis* [68] (Figure S8). The *gpr* gene of *B. subtilis* codes for a spore-specific protease required for degradation of the DNA-protecting small acid-soluble spore proteins (SASP) during spore germination ([1]; see also below). Even though this gene is under the dual control of σ^F^ and σ^G^ in *B. subtilis*, the *C. difficille* orthologue of *gpr* (CD2470) was chosen as a reporter for σ^F^ activity (Figure S8A). First, qRT-PCR showed that transcription of the *C. difficille* orthologue (CD2470) was severely reduced in a *sigF* mutant (Figure 6A). Secondly, expression of a P*_gpr_*-*SNAP^Cd^* fusion, monitored by fluorescence microscopy, was confined to the forespore and detected soon after asymmetric division in 66% of the cells that were at this stage of sporulation (Figure 6B). Lastly, expression was eliminated by disruption of the *sigF* gene but detected in 99% of the cells of the *sigG* mutant (compared for the wild type at the same stage, *i.e.*, 95%) (Figure 6B). This suggests that σ^G^ does not contribute significantly to *gpr* expression. Forespore-specific expression of P*_gpr_*-*SNAP^Cd^* was also detected following engulfment completion (Figure 6B). Therefore, in spite of expression of the *sigF* gene in both the forespore and the mother cell, σ^F^ is active exclusively in the forespore. In these experiments, all of the SNAP protein produced from P*_gpr_*-*SNAP^Cd^* was labeled with the TMR-Star substrate (Figure 7A). A quantitative analysis of the fluorescence signal from P*_gpr_*-*SNAP^Cd^* showed no significant difference between cells before (average signal for classes *a*+*b*, 2.0±0.5) or after engulfment completion (classes *c*+*d*, 1.9±0.7) (Figure 7B). This suggests that σ^F^ is active in the forespore throughout development.

To monitor the activity of σ^E^, we examined expression of the first gene, *spoIIIAA*, of the *spoIIIA* operon. This operon is under the control of σ^E^ in *B. subtilis* [69]–[71] and sequences that conform well to the consensus for promoter recognition by *B. subtilis* σ^E^ are found just upstream of the *C. difficile spoIIIAA* gene (or CD1192) (Figure S8B). The qRT-PCR experiments showed that expression of *spoIIIAA* was much more severely affected by a mutation in *sigE* than by disruption of *sigF* (Figure 6A). While consistent with a direct control of *spoIIIAA* by σ^E^, this observation adds to the evidence suggesting that unlike in *B. subtilis* [21], [66], [67], the activity of σ^E^ is at least partially independent on the prior activation of σ^F^ (as also hinted by the observation that transcription of the *sigK* gene, abolished by mutation of *sigE*, was still detected in a fraction of cells of a *sigF* mutant; above). If so, then expression of a P*_spoIIIAA_*-*SNAP^Cd^* fusion should be confined to the mother cell, dependent on *sigE*, but partially independent on *sigF*. P*_spoIIIAA_*-*SNAP^Cd^*-driven SNAP production was indeed confined to the mother cell, detected just after asymmetric division in 89% of the cells scored at this stage of sporulation, eliminated by mutation of *sigE*, but still detected (in the mother cell) in 21% of *sigF* cells (Figure 6B). Labeling of the SNAP protein produced from the P*_spoIIIAA_*-*SNAP^Cd^* fusion was quantitative (Figure 7A), and the quantitative analysis of the average fluorescence signal shows no significant difference in expression levels before or after engulfment completion (Figure 7B). P*_spoIIIAA_*-*SNAP^Cd^* expression persisted until late stages in development, and was still detected for cells in which phase bright spores were seen (Figure 7B).

### Requirements for the activity of σ^G^ and σ^K^

The *sspA* gene of *B. subtilis* codes for a small acid-soluble spore protein (SASP) that, together with other SASP family members, binds to and protects the spore DNA [1]. Expression of *sspA* in *B. subtilis* is controlled by σ^G^ [68], [69], and a σ^G^-type promoter can be recognized upstream of the *C. difficile* orthologue (CD2688) (Figure S8C). Unexpectedly, a mutation in *sigF* caused a greater decrease in *sspA* transcription than disruption of *sigE* or *sigG*, in our qRT-PCR analysis (Figure 6A). While not excluding a contribution of σ^F^ to the expression of *sspA*, this result may be affected by the lack of synchronization of sporulation in the liquid SM cultures. Consistent with σ^G^ control of *sspA* in *C difficile*, P*_sspA_*-driven SNAP production was confined to the forespore and eliminated by disruption of *sigF* or of *sigG* (but not of *sigE* or *sigK*) (Figure 6B). This is in agreement with the requirement for σ^F^ for the transcription of *sigG* (above), and seems to exclude a contribution of σ^F^ for *sspA* transcription as suggested by the qRT-PCR analysis. *sspA* expression was detected in 50% of the cells that had just completed asymmetric division, but also throughout the engulfment sequence (72% of the cells), following engulfment completion (85% of the cells scored), and in cells (75%) carrying phase bright spores (Figure 6B). Our analysis of *sigG* transcription suggested that it increased following engulfment completion, with a stronger auto-regulatory component than in pre-engulfed cells (above). In *B. subtilis*, continued transcription in the forespore when (upon engulfment completion) it becomes isolated from the surrounding medium, requires the activity of σ^E^ [72]–[76]. To determine whether the activity of σ^G^ increased following engulfment completion in a manner that required σ^E^, we quantified the SNAP-TMR signal in cells expressing P*_sspA_*-*SNAP^Cd^*. Control experiments showed that all the SNAP protein produced from the P*_sspA_*-SNAP^Cd^ fusion was labeled with the TMR-Star substrate (Figure 7A). The average intensity of the SNAP-TMR signal increased from 1.6±0.5 before engulfment completion (classes *a*+*b*) to 2.1±04, following engulfment completion (classes *c*+*d*) (p<0.0001) (Figure 7B). This result is consistent with the analysis of *sigG* transcription (above) and indicates that the activity of σ^G^ increases following engulfment completion. Importantly, even though *sspA* expression was found for 61% of the *sigE* mutant cells, disruption of *sigE* reduced the average fluorescence signal in the forespore (1.6±0.7) to the level seen before engulfment completion for the wild type (1.6±0.5). We conclude that disruption of *sigE* does not prevent activity of σ^G^ prior to engulfment completion.

Finally, to monitor the activity of σ^K^, we examined expression of the *cotE* gene, coding for an abundant spore coat protein in *C. difficile* [55]. This gene has no counterpart in *B. subtilis*, but as shown above, production of a CotE-SNAP translational fusion was dependent on σ^K^ (Figure 2E) and a sequence that conforms well to the consensus for σ^K^ promoters of *B. subtilis* can be recognized in its promoter region (Figure S8D). qRT-PCR experiments show that disruption of the *sigE* and *sigK* genes caused a much stronger reduction in the expression of *cotE* than mutations in *sigF* or *sigG* (Figure 6A). While not excluding a contribution from σ^E^, the qRT-PCR data are in line with the interpretation that the main regulator of *cotE* expression is σ^K^ (with σ^E^ driving production of σ^K^). We note that the reduced effect of the *sigF* mutation on *cotE* expression is in agreement with the view that σ^E^ production is partially independent on σ^F^, as discussed above. We also note that the reduced effect of the *sigG* mutation on *cotE* expression is in agreement with the morphological analysis and the data on the assembly of the CotE-SNAP fusion (Figure 2D and E), suggesting σ^K^-dependent deposition of coat material independently of σ^G^. Fluorescence microscopy reveals that expression of P*_cotE_*-SNAP^Cd^ is confined to the mother cell (Figure 6B). However, expression of P*_cotE_*-SNAP^Cd^ was found just after asymmetric division in only 2% of the cells, and during engulfment in only 10% of the cells (Figure 6B). Expression increased to 66% of the cells after engulfment completion, and to 88% of the cells that showed phase bright spores (Figure 6B). Expression of P*_cotE_*-*SNAP^Cd^* was eliminated by disruption of *sigE*, but retained in 3% of the sporulating cells of a *sigF* mutant (Figure 6B). This is consistent with data presented above, also in line with the inference that the activity of σ^K^ is partially independent on *sigF* (Figure 6A and B). Moreover, 66% of the cells of a *sigG* mutant that had completed the engulfment process (as illustrated in Figure 6B) showed expression of the reporter fusion, again suggesting σ^K^ activity independently of σ^G^. Interestingly, disruption of *sigK* did not abolish expression of the fusion, which was detected in 90% of the sporulating cells, but at low levels (Figure 6B). This raises the possibility that σ^E^ is responsible for the few cells that produce the reporter prior to engulfment completion.

To test these possibilities quantitatively, we first verified that all the SNAP-tag produced from the P*_cotE_*-*SNAP^Cd^* fusion was labeled, under our experimental conditions (Figure 7A). The average intensity of the SNAP-TMR signal was of 0.6±0.1 for cells of the wild type strain prior to engulfment completion (classes *a*+*b*), of 1.2±0.8 for those that had just completed engulfment (class *c*), and of 3.3±1.1 for cells with phase bright spores (class *d*) (Figure 7B). Inactivation of *sigG* did not affect the expression level of the fusion prior to engulfment completion (classes *a*+*b* for the *sigG* mutant, average signal, 0.6±0.1), nor did it prevent expression following engulfment completion (class *c* of the *sigG* mutant, 1.3±0.8) (Figure 7B). However, the average fluorescence signal for all classes of the *sigG* mutant (1.3±0.8) is significantly lower than the average for all classes of the wild type (1.9±1.3) (p<0.001) (Figure 7B). Finally, the average fluorescence signal for all cells of the *sigK* mutant was lower (0.3±0.1) than for pre-engulfment cells of the wild type (classes *a*+*b*, 0.6±0.1) (p<0.0001), suggesting that both σ^E^ and σ^K^ contribute to expression of the reporter fusion in these cells. Together, these data suggest that the main period of σ^K^ activity is delayed relative to engulfment completion, and coincides with development of spore refractility.

## Discussion

In this work, we analyzed the function of the four cell type-specific sigma factors of sporulation in *C. difficile*, and we studied gene expression in relation to the course of spore morphogenesis. The morphological characterization of mutants for the *sigG* genes allowed us to assign functions and to define the main periods of activity for the 4 cell type-specific sporulation sigma factors. In addition, the use of a fluorescence transcriptional reporter for single cell analysis enabled us to establish the time, cell type and dependency of transcription of the *sig* genes, as well as the time and requirements for activity of the four cell type-specific sigma factors.

### Transcription of *sigF* and *sigE*, and activity of σ^F^ and σ^E^

The cytological and TEM analysis shows that the *sigF* and *sigE* mutants are arrested just after asymmetric division. It follows that σ^F^ and σ^E^ control early stages of development in *C. difficile*, consistent with the function of these sigma factors in *B. subtilis*. Disruption of *sigE* also arrested development just after asymmetric division in *C. perfringens* [29]. In contrast, disruption of either the *sigF* or *sigE* genes in *C. acetobutylicum* blocks sporulation prior to asymmetric division [31], [33]. In *C. difficile*, expression of both *sigF* and *sigE* commenced in predivisional cells, in line with work showing that expression of the *sigF*-containing operon (also coding for two other proteins, SpoIIAA and SpoIIAB, that control σ^F^) occurs from a σ^H^ and Spo0A-controlled promoter, and with the observation that transcription of *sigE* is activated from a σ^A^-type promoter to which Spo0A also binds [60], [61]. In *B. subtilis*, following asymmetric septation, Spo0A becomes a cell-specific transcription factor, active predominantly in the mother cell [77]. This may also be the case in *C. difficile*, because transcription of *sigE* increased in the mother cell, relative to the forespore, following asymmetric division (Figure 7B).

In *B. subtilis*, σ^F^ is held in an inactive complex by the anti-sigma factor SpoIIAB [21], [23]. The reaction that releases σ^F^ takes place specifically in the forespore, soon after septation, and involves the anti-anti sigma factor SpoIIAA and the SpoIIE phosphatase. SpoIIAB, SpoIIAA and SpoIIE are produced in the *C. difficile* predivisional cell under Spo0A control [24]–[27], [60], [61]. Because the activity of σ^F^ was confined to the forespore, we presume that the pathway leading to the forespore-specific activation of this sigma factor is also conserved. In *C. acetobutylicum*, this pathway may lead to σ^F^ activation in pre-divisional cells, as disruption of *sigF* or *spoIIE* blocks sporulation prior to asymmetric division [33], [35].

In *B. subtilis*, σ^E^ is also synthesized in the predivisional but as an inactive pro-protein [21], [23]. Processing of pro-σ^E^ in the mother cell requires activation of the SpoIIGA protease by SpoIIR, a σ^F^-controlled signaling protein secreted from the forespore [66], [67]. Hence, the activity of σ^E^ requires the prior activation of σ^F^. In *C. difficile*, the activity of σ^E^ was also restricted to the mother cell (Figure 7B). Because σ^E^ of *C. difficile* bears, like its *B. subtilis* counterpart, a pro sequence, and because the SpoIIGA protease and SpoIIR are conserved [24]–[27], the σ^E^ activation pathway also seems conserved. Strikingly however, both the qRT-PCR and the SNAP labeling experiments showed that the activity of σ^E^ is at least partially independent on σ^F^ (Figure 6). We do not know whether production of SpoIIR is also partially independent on σ^F^. However, in *C. acetobutylicum*, in which σ^F^ is activated (and required) prior to asymmetric septation [33], [35], production of SpoIIR is, at least in part, independent of σ^F^ [33].

### Production and activity of σ^G^

The cytological and TEM analysis showed that a *sigG* mutant completes the engulfment sequence, suggesting that σ^G^ is mainly required for late stages in development, consistent with its role in *B. subtilis*. Disruption of *sigG* also causes a late morphological block in *C. acetobutylicum* [78]. In *B. subtilis*, the forespore-specific transcription of *sigG* is initiated by σ^F^ but is delayed, relative to other σ^F^-dependent genes, towards the engulfment sequence [21], [64], [68], [69]. Moreover, the activity of σ^E^, in the mother cell, is required for transcription of *sigG* [21], [64]. In contrast, transcription of *sigG* in *C. difficile*, was detected soon after asymmetric septation, and was not dependent on σ^E^ (Figure 4 and 5). Transcription of *sigG* also appears to be independent of *sigE* in *C. perfringens* [29]. The main period of *sigG* transcription in *B. subtilis* relies on an auto-regulatory loop activated coincidently with engulfment completion [65]. Therefore, the main period of σ^G^ activity coincides with engulfment completion. At least the anti-sigma factor CsfB (σ^F^-controlled) appears important for impeding the σ^G^ auto-regulatory loop from functioning prior to engulfment completion, the main period of activity of the preceding forespore sigma factor, σ^F^ [21], [23], [72], [79], [80]. CsfB is absent from *C. difficile* as well as from other Clostridia [24], [25], [27]. In *C. difficile*, not only is transcription of *sigG* observed soon after asymmetric division, but the activity of σ^G^, is also detected prior to engulfment completion. Nevertheless, our analysis indicates that σ^G^ activity increases following engulfment completion. In addition, our results suggest that σ^G^ is auto-regulatory both before, and more markedly, following engulfment completion.

A universal feature of endosporulation is the isolation of the forespore, surrounded by two membranes, from the external medium at the end of the engulfment sequence. In *B. subtilis*, the 8 mother cell proteins encoded by the *spoIIIA* operon, which localize to the forespore outer membrane, and the forespore-specific SpoIIQ protein, which localizes to the forespore inner membrane, are involved in the assembly of a specialized secretion system that links the cytoplasm of the two cells [72]–[76]. Recent work has shown that the SpoIIIA-SpoIIQ secretion system functions as a feeding tube required for continued macromolecular synthesis in the engulfed forespore [76]. Mutation of *sigE* reduced the activity of σ^G^ but because the mutant is blocked at an early stage, we do not presently know whether σ^E^ is required for σ^G^ activity in the engulfed forespore. The SpoIIIAH and SpoIIQ proteins also facilitate forespore engulfment in *B. subtilis* [81]. The *spoIIIA* operon is conserved in sporeformers [24], [25], [27], and *spoIIIA* is under σ^E^ control in *C. difficile* (this work). A gene, CD0125, coding for a LytM-containing protein (as the *B. subtilis* SpoIIQ protein) may represent a non-orthologous gene replacement of *spoIIQ* [24]. We do not yet know whether *spoIIIA* and CD0125 are essential for sporulation in *C. difficile* and if so, whether they are required for engulfment and/or continued gene expression in the engulfed forespore.

### Production and activity of σ^K^

The TEM analysis shows that the *sigK* mutant of *C. difficile* lacks a visible coat (Figure 2D). However, as in *B. subtilis* [15], [16] assembly of the coat begins with σ^E^, as suggested by the forespore localization of CotB-SNAP in cells of the *sigK* mutant, and supported by recent work on the analysis of coat morphogenetic proteins SpoIVA and SipL [42]. Most likely, σ^K^ controls the final stages in the assembly of the spore surface structures, including the coat and exosporium. However, σ^K^ is not a strict requirement for the formation of heat resistant spores (Figure 2 and Table 1), and we presume that σ^E^ and σ^G^ (see above) are largely responsible for synthesis of the spore cortex. Final assembly of the coat together with the role of *C. difficile* σ^K^ in mother cell autolysis, are functions shared with its *B. subtilis* counterpart.

Transcription and activity of the *C. difficile sigK* gene was dependent on *sigE*, and was detected at low levels prior to engulfment completion. However, both transcription and activity increased, following engulfment completion, coincidently with the appearance of phase grey and phase bright spores. Transcription of the *sigK* and *spoIVCA* genes of *B. subtilis*, the latter coding for the recombinase that excises the *skin* element, is initiated under the control of σ^E^ with the assistance of the regulatory protein SpoIIID, and is delayed relative to a first wave of σ^E^-directed genes [21], [23], [69], [70]. SpoIIID is conserved in *C. difficile* [27] and it may only accumulate to levels sufficient to enhance *sigK* and *spoIVCA* transcription at late stages in morphogenesis. Two observations highlight the importance of the *skin* element in *C. difficile*. First, with the exception of an asporogenous strain of *C. tetani*, the *skin* element is not present in other Clostridial species [17], [39]. Second, not only a *skin*-less allele of *sigK* fails to complement a *sigK* mutation but also acts as a dominant negative mutation [39], blocking entry into sporulation (Figure 3D and E) (while these results seem to imply that σ^K^ is auto-regulatory, we did not detect auto-regulation of *sigK* in our single cell analysis). Absence of the *skin* element may allow the recruitment of σ^K^ for other functions. In *C. perfringens* and in *C. botulinum*, σ^K^ is produced in pre-divisional cells, and is involved in enterotoxin production in the first, and in cold and osmotic stress tolerance in the second [29], [82].

A key finding of the present study is that contrary to *B. subtilis*, *sigG* is not essential for the activity of σ^K^. In *B. subtilis* a signaling protein, SpoIVB, secreted from the forespore activates the pro-σ^K^ processing protease SpoIVFB, which is kept inactive in a complex with BofA and SpoIVFA, embedded in the forespore outer membrane [21], [22]. SpoIVFB, BofA and possibly also SpoIVFA are absent from *C. difficile*, suggesting that the σ^G^ to σ^K^ pathway is absent and consistent with the lack of a pro-sequence [24], [27], [36]. However, *C. difficile* codes for two orthologues of SpoIVB [24], [25], [27]. Mutations that bypass the need for *sigG* or *spoIVB* in *B. subtilis* result in coat deposition, but not cortex formation, phenocopying the *sigG* mutant of *C. difficile* [50]. In *B. subtilis*, SpoIVB is also required for the engulfment-regulated proteolysis of SpoIIQ [83]. The *C. difficile* SpoIVB orthologues may be involved in cortex formation and/or proteolysis of CD0125 (above).

While the activity of σ^K^ did not require σ^G^, our data shows that mutation of *sigG* reduced the activity of σ^K^ at late stages of spore morphogenesis. Because the *sigG* mutant fails to form phase grey/bright spores, we do not presently know if a forespore-mother cell signaling operates at this stage, or whether the late stages in spore morphogenesis serve as a cue for enhanced activity of σ^K^.

### Concluding remarks

**Figure 8:**
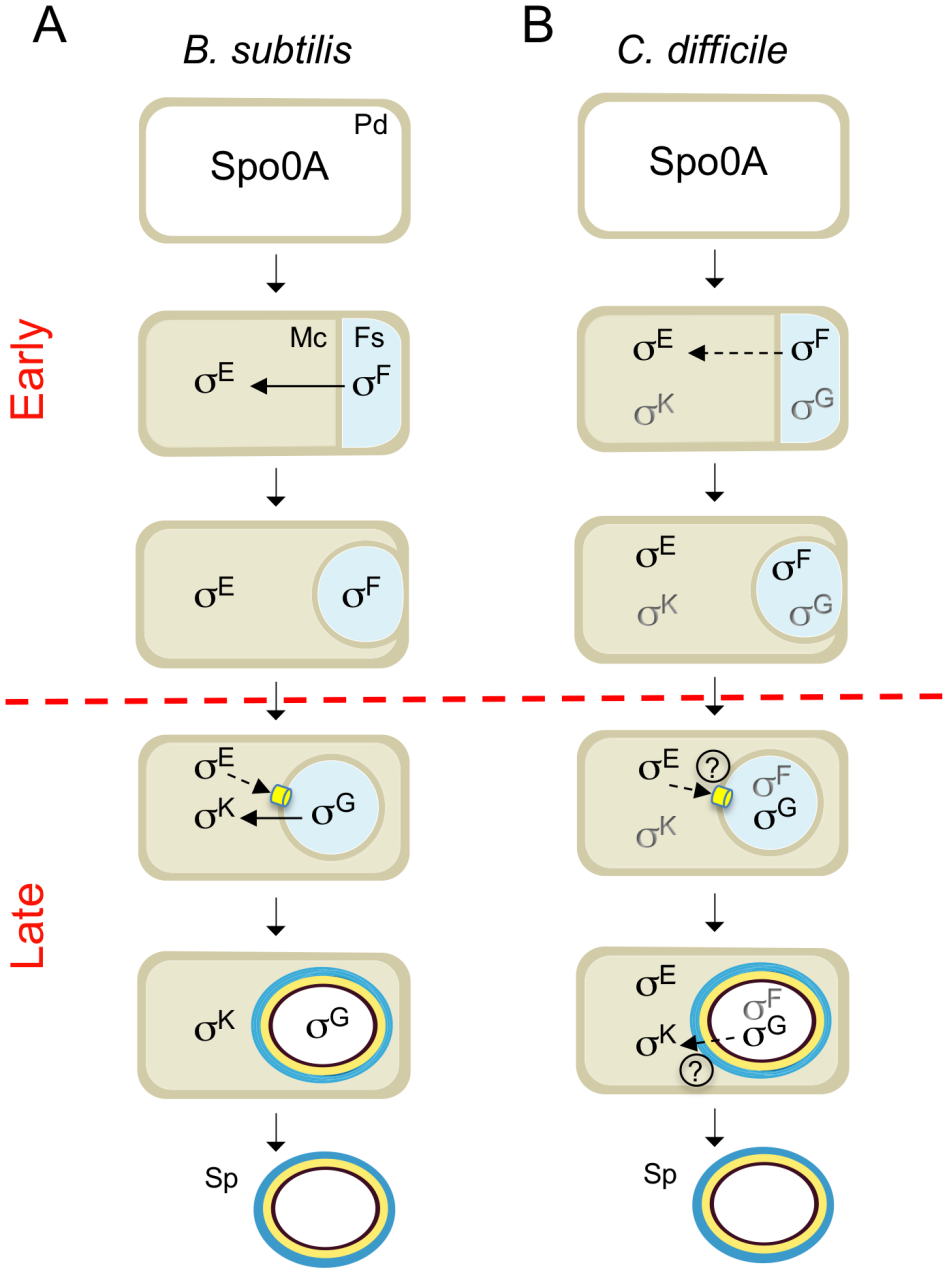
Stages and cell of σ^F^, σ^E^, σ^G^ and σ^K^ activity. The figure compares the main periods of activity of the 4 cell type-specific sigma factors of sporulation in *B. subtilis* (A) and *C. difficile* (B). The figure incorporates data on the morphological analysis of sporulation in the *sigF*, *sigE*, *sigG* and *sigK* mutants (Figure 2, 4 and 6) and on the stage, dependencies and cell where σ^F^, σ^E^ , σ^G^ and σ^K^ are active. Solid or broken arrows represent dependencies or partial dependencies, respectively. The representation of the *C. difficile* sigma factors indicates activity; black indicates the main period of activity. Possible cell-cell signaling pathways are show by both a broken line and a question mark. The SpoIIIA-SpoIIQ/CD0125 channel is represented in yellow. PD: predivisional cell; MC: mother cell; FS: forespore. The red horizontal broken line distinguishes early (prior to engulfment completion) from late (post-engulfment completion) development.

We show that the main periods of activity of the four cell type-specific sigma factors of *C. difficile* are conserved, relative to the *B. subtilis* model, with σ^F^ and σ^E^ controlling early stages of development and σ^G^ and σ^K^ governing late developmental events (Figure 8A and B). However, the fact that the activity of σ^E^ was partially independent of σ^F^, and that σ^G^ or σ^K^ did not require σ^E^ or σ^G^, respectively, seems to imply a weaker connection between the forespore and mother cell lines of gene expression. In spite of the important differences in the roles of the sporulation sigma factors and the regulatory circuits leading to their activation (Figure 8A and B), overall, in what concerns the genetic control of sporulation, *C. difficile* seems closer to the model organism *B. subtilis* than the other Clostridial species that have been studied. Differences in the function/period of activity of the sporulation sigma factors in other Clostridial species, may be related to the coordination of solventogenesis, toxin production or other functions with sporulation [28]–[35]. We note however that the relationship between toxinogenesis and spore formation in *C. difficile* is still unclear [13], [60].

Together with the accompanying work of Saujet and co-authors [62], our study provides the first comprehensive description of spore morphogenesis in relation to cell type-specific gene expression in a Clostridial species that is also an important human pathogen. The two studies establish a platform for analyzing the control of toxin production in relation to *C. difficile* sporulation, and for the functional characterization of genes predicted to be important for spore functions related to host colonization, spore germination, recurrent sporulation in the host, and spore dissemination.

## Materials and Methods

### Strains and general techniques

Bacterial strains and their relevant properties are listed in Table S2. The *Escherichia coli* strain DH5α (Bethesda Research laboratories) was used for molecular cloning. Luria-Bertani medium was routinely used for growth and maintenance of *E. coli* and *B. subtilis*. The *B. subtilis* strains are congenic derivatives of the Spo^+^ strain MB24 (*trpC2 metC3*). Sporulation of *B. subtilis* was induced by growth and exhaustion in Difco sporulation medium (DSM) [84]. When indicated, ampicillin (100 µg/ml) or chloramphenicol (15 µg/ml) was added to the culture medium. The *C. difficile* strains used in this study are congenic derivatives of the wild type strain 630Δ*erm* [85] and were routinely grown anaerobically (5% H2, 15% CO2, 80% N2) at 37°C in brain heart infusion (BHI) medium (Difco), BHIS [BHI medium supplemented with yeast extract (5 mg/ml) and L-cysteine (0.1%), or SM medium (for 1l: 90 g Bacto-tryptone, 5 g Bacto-peptone, 1 g (NH_4_)_2_SO_4_ and 1.5 g Tris base)] [41]. Sporulation assays were performed in SM medium [41]. When necessary, cefoxitin (25 µg/ml), thiamphenicol (15 µg/ml), or erythromycin (5 µg/ml) was added to *C. difficile* cultures.

### Sporulation assays

Overnight cultures grown at 37°C in BHI were used to inoculate SM medium (at a dilution of 1∶200). At specific time points, 1 ml of culture was withdrawn, serially diluted in phosphate-buffered saline (PBS; 137 mM NaCl, 10 mM Phosphate, 2.7 mM KCl, pH 7.4), and plated before and after heat treatment (30 min at 60°C), to determine the total and heat-resistant colony forming units (CFU). The samples were plated onto BHI plates supplemented with 0.1% taurocholate (Sigma-Aldrich), to promote efficient spore germination [41]. The percentage of sporulation was determined as the ratio between the number of spores/ml and the total number of bacteria/ml times 100.

### RNA isolation and real-time quantitative RT-PCR

In a preliminary set of experiments, we defined the time for which the difference in expression of a selected σ target gene between the wild type and the corresponding mutant strain was highest. To study σ^E^- or σ^F^-dependent control, we harvested cells from 630Δ*erm*, *sigF* and *sigE* mutants after 14 h of growth in SM medium. Strain 630Δ*erm* and the *sigG* or the *sigK* mutants were harvested after 19 h (630Δ*erm*, *sigG* mutant) and 24 h (630Δ*erm*, *sigK* mutant) of growth in SM medium. Total RNA was extracted from at least two independent cultures. After centrifugation, the culture pellets were resuspended in RNApro solution (MP Biomedicals) and RNA extracted using the FastRNA Pro Blue Kit, according to the manufacturer's instructions. The RNA quality was determined using RNA 6000 Nano Reagents (Agilent). For quantitative RT-PCR experiments, 1 µg of total RNA was heated at 70°C for 10 min along with 1 µg of hexamer oligonucleotide primers p(dN)_6_ (Roche). After slow cooling, cDNAs were synthesized as previously described [61]. The reverse transcriptase was inactivated by incubation at 85°C for 5 min. Real-time quantitative RT-PCR was performed twice in a 20 µl reaction volume containing 20 ng of cDNAs, 10 µl of FastStart SYBR Green Master mix (ROX, Roche) and 200 nM gene-specific primers in a AB7300 real-time PCR instrument (Applied Biosystems). The primers used for each marker are listed in Table S3. Amplification and detection were performed as previously described [61]. In each sample, the quantity of cDNAs of a gene was normalized to the quantity of cDNAs of the DNApolIII gene. The relative transcript changes were calculated using the 2^−ΔΔCt^ method as described [61].

### Transcriptional and translational *SNAP^Cd^* fusions

The construction of transcriptional fusions of the promoters for the *sigF*, *sigE*, *sigG* and *sigK* genes, as well as the construction of translational fusions of *cotB* and *cotE* to the SNAP-tag [86] is described in detail in Text S1. In these plasmids, listed in Table S4, we used a synthetic SNAP cassette, codon usage optimized for *C. difficile* (DNA 2.0, Menlo Park, CA), which we termed *SNAP^Cd^* (the sequence is available for download at www.itqb.unl.pt/~aoh/SNAPCdDNAseq.docx).

### SNAP labeling and analysis

Whole cell extracts were obtained by withdrawing 10 ml samples from *C. difficile* cultures in brain heart infusion (BHI) for the P*_tet_*-SNAP-bearing strains, or in SM medium for the sporulation experiments, at the desired times. The extracts were prepared immediately following labeling with 250 nM of the TMR-Star substrate (New England Biolabs), for 30 min in the dark. Following labeling, the cells were collected by centrifugation (4000×g, for 5 min at 4°C), the cell sediment was washed with phosphate-buffered saline (PBS) and resuspended in 1 ml French press buffer (10 mM Tris pH 8.0, 10 mM MgCl2, 0.5 mM EDTA, 0.2 mM NaCl, 10% Glycerol, 1 mM PMSF). The cells were lysed using a French pressure cell (18000 lb/in^2^). Proteins in the extracts were resolved on 15% SDS-PAGE gels. The gels were first scanned in a Fuji TLA-5100 fluorimager, and then subject to immunoblot analysis as described before [65]. The anti-SNAP antibody (New England Biolabs) was used at a 1∶1000 dilution, and a rabbit secondary antibody conjugated to horseradish peroxidase (Sigma) was used at dilution 1∶10000. The immunoblots were developed with enhanced chemiluminescence reagents (Amersham Pharmacia Biotech).

### Microscopy and image analysis

Samples of 1 ml were withdrawn from BHI or SM cultures at the desired times following inoculation, and the cells collected by centrifugation (4000×g for 5 min). The cells were washed with 1 ml of PBS, and ressuspended in 0.1 ml of PBS supplemented with the lipophilic styryl membrane dye *N*-(3-triethylammoniumprpyl)-4-(*p*-diethylaminophenyl-hexatrienyl) pyridinium dibromide (FM4-64; 10 µg.ml^−1^) [43], [87], and the DNA stain DAPI (4′,6-diamidino-2-phenylindole; 50 µg.ml^−1^) (both from Molecular Probes, Invitrogen). For the live/dead assay, samples were collected as described above, ressuspended in 0.05 ml of PBS and mixed with an equal volume of 2× LIVE/DEAD BacLight 2× staining reagent mixture (Molecular Probes, Invitrogen) containing Propidium iodide (30 µM final concentration) and syto9 (6 µM final concentration).

For SNAP labeling experiments, cells in culture samples were labeled with TMR-Star (as above), collected by centrifugation (4000×g, 3 min, at room temperature), washed four times with 1 ml of PBS, and finally ressuspended in 1 ml of PBS containing the membrane dye Mitotracker Green (0.5 µg.ml^−1^) (Molecular Probes, Invitrogen).

For phase contrast and fluorescence microscopy, cells were mounted on 1.7% agarose coated glass slides and observed on a Leica DM6000B microscope equipped with a phase contrast Uplan F1 100× objective and a CCD Ixon camera (Andor Technologies) [65]. Images were acquired and analyzed using the Metamorph software suite version 5.8 (Universal Imaging), and adjusted and cropped using ImageJ (http://rsbweb.nih.gov/ij/). Exposure times were adjusted and defined for each SNAP transcriptional or translational fusion analyzed. For quantification of the SNAP^Cd^ signal resulting from transcriptional fusions, 6×6 pixel regions were defined in the desired cell and the average pixel intensity was calculated, and corrected by subtracting the average pixel intensity of the background. Small fluctuations of fluorescence among different fields were corrected by normalizing to the average pixel intensity obtained for the intrinsic autofluorescence of *C. difficile* cells [88].

### Statistical analysis

Statistical analysis was carried out using GraphPad Prism (Version 6.0; GraphPad Software Inc.). The non-parametric Kolmogorov-Smirnov test (KS-test) was applied to compare distributions obtained from quantifications of the SNAP-TMR signal. The P-value is indicated for all comparisons whose differences were found to be statistically significant. Although the results presented are from a single experiment, all experiments involving quantification of a fluorescence signal were performed independently three times and only results that were considered statistically significant by a KS-test in all three experiments were considered to be statistically relevant.

### Electron microscopy

For transmission electron microscopy electron (TEM) analysis, cells of the wild type 630Δ*erm* strains and of the various *sig* mutants were collected at various times following inoculation onto Columbia Horse Blood Agar plates (BioMérieux). The high fraction of sporulating cells under these growth conditions, facilitates the TEM analysis [89]. Samples were processed for TEM as described previously [90].

## Supporting Information

Figure S1 Sporulation of strain 630Δ*erm* in SM (red), BHI (dark blue) and BHIS (light blue) liquid media. The heat resistant spore titer (filled symbols) and the total cell titer (open symbols) was measured for culture samples collected at the indicated time points. Values represent the average ± SD of three independent experiments.
(EPS)

Figure S2 *C. difficile* spore structure. (A) Differential staining of *C. difficile* and *B. subtilis* spores by the membrane dye FM4-64. Sporulating cells (upper panel) and free spores (lower panel) of *C. difficile* 630Δ*erm* strain were collected and mixed with sporulating cells or free spores of *B. subtilis* MB24, respectively. Sporulating cells and free spores of *C. difficile* 630Δ*erm* were collected after 2 and 5 days of growth in BHI agar. *B. subtilis* MB24 cells were collected 6 and 24 hours after the onset of sporulation in DSM. After mixing, cells were stained with FM4-64, and analyzed by phase contrast (PC) and fluorescence microscopy. FITC column shows the typical autofluorescence of *C. difficile* cells. The *B. subtilis* strain used produces a CFP fusion to a coat protein (Tgl-CFP). Scale bar, 1 µm. (B) Staining of *Bacillus cereus* strain ATCC4342 sporulating cells and free spores with FM4-64. *B. cereus* cells were imaged after 48 h of growth in Leighton-Doi medium at 30°C. Scale bar, 1 µm. (C) Decoating of *C. difficile* spores drastically reduces staining with FM4-64. *C. difficile* purified spores were submitted or not to a decoating treatment (see Text S1) prior to staining with FM4-64, and analysed by fluorescence microscopy. The percentage of total spores labeled with FM4-64 was scored for both intact and decoated spores (total number of spores scored (n) is indicated). Scale bar, 1 µm.
(EPS)

Figure S3 Ultrastructure of 630Δ*erm* spores. (A) and (B) show a spore within a lysed mother cell; (C) and (D) show a free spore; (B) and (D) are a magnification of the region encircled in (A) and (C). Arrows point to a layer apposed to the external surface of the spore. The main structural features of *C.difficile* spores are indicated in C/D: Cr, core; Cx, cortex; IC, inner coat; OC, outer coat. Scale bar, 1 µm (A,C) or 0.5 µm (B,D).
(EPS)

Figure S4 Inactivation of the *sigF*, *sigE*, *sigG* and *sigK* genes in *C. difficile* using the ClosTron system. (A) Genetic organisation of the *C. difficile* chromosome in the vicinity of *sigF*, *sigE*, *sigG* and *sigK* (interrupted by the 14.2 kbp *skin* element). The red arrow indicates the point of insertion of the re-targeted type II introns used for gene disruption. The extent of the DNA fragment present in the indicated replicative plasmids used for in trans complemetation of the insertional mutations is shown below each of the genetic maps, except for the *sigK* gene (see Figure 3C). (B) Schematic representation of gene inactivation by a type II Intron with an associated RAM. The group II intron (bracket), originally in pMTL007 (top), carries a RAM element (yellow) interrupting an *ermB* determinant (blue). The intron was retargeted to the sig gene of interest (black; middle) by altering the IBS, EBS1 and EBS2 sequences (grey and white stripes; top) by overlapping PCR. Splicing out of the td group I intron from the *ermB* gene in the RAM restores a functional marker allowing positive selection of mutants following intron integration. Primers used to confirm the integration and orientation of the type II intron are also indicated (bottom). (C, D) Chromosomal DNA of Em^R^ *C. difficile* conjugants was screened by PCR using primer pairs RAM-F/R to confirm splicing out of the group I intron (C), or with primer pairs sigFw/Rev, sigFw/EBS and RAM-F/sigRev to confirm disruption of the *sig* genes (D). pMTL007 (C) and chromosomal DNA from the 630Δ*erm* (wild type, wt) strain (D) were used as controls. (E) Southern blot of HindIII-digested genomic DNA from *C. difficile* 630Δ*erm* (wild type, wt), *sigF*, *sigE*, *sigG* and *sigK* mutant strains. The probe used corresponds to part of the intron sequence (see Suplemental Materials and Methods). The position of DNA size markers is indicate on the left side of the panel.
(EPS)

Figure S5 SNAP-tag vectors used in the study. (A) Schematic representation of pFT46, a plasmid carrying *SNAP^Cd^* under the control of the P*_tet_* inducible promotor (darker blue). The P*_tet_* -*SNAP^Cd^* fusion is flanked by the transcriptional terminator sites of the *slpA* and *fdx* genes. Unique restricition sites are indicated. (B) Schematic representation and features of pFT47 vector, a derivative of pMTL84121 [45], coding for a codon usage-optimized SNAP-tag (*SNAP^Cd^*). Unique restriction sites (SbfI, AscI, FseI, PmeI) that allow replacement of functional blocks among plasmids of the pMTL80000 series are also indicated along the circular plasmid map. A detailled representation of the region between XhoI/BamHI and HindIII sites (in blue) is shown on the bottom (for pFT47) and in (C) (for pFT58). The *SNAP^Cd^* gene, preceded by an RBS, was inserted between the XhoI and HindIII sites within the multiple cloning site (MCS) of pMTL84121 (unique sites upstream of XhoI, that are part of the pMTL84121 MCS, are indicated), yielding pFT47, a vector suitable for transcriptional fusions. (C) For translational fusions, the region between BamHI and HindIII sites of pFT47 (in blue) was replaced by the one depicted, yielding pFT58. This vector retains the main features of pFT47, except that a linker preceeds the SNAP coding sequence, from which the start codon was also removed, allowing the construction of C-terminal translational fusions to SNAP. NB: Only the 5 last codons of the linker sequence are present in pFT58 (see Materials and Methods).
(EPS)

Figure S6 The SNAP-tag as a fluorescent reporter in *C. difficile*. (A) Fluorescence microscopy of *C. difficile* cells producing SNAP under the control of P*_tet_* (in pFT46). Cells were grown in BHI liquid medium and induced with anhydrous tetracycline (ATc) at the mid-exponential phase, during 1 hour. Cells were then labeled with for 30 minutes with the indicated concentrations (nM) of the SNAP substrate TMR-Star and examined by phase contrast (PC) and fluorescence microscopy, to monitor SNAP production. The panels on the right show the relative levels of SNAP-TMR-Star fluorescence within individual cells (n = 150), for the indicated labeling times. Scale bar, 1 µm. (B) Values represented by the bars are the average ± SD of the fluorescence intensity shown in A, for each of the labeling concentrations of TMR-Star used. (C) Percentage of cells showing a fluorescent signal above the background level for each labeling concentration. (D) Whole cell extracts were prepared from strain 630Δ*erm* carrying pFT46 grown until mid-exponential phase, time at which cells were induced by the addition of ATc (250 ng/ml), for 1 hour. The extracts were prepared immediately following labeling with increasing concentrations of TMR-Star, as described in (A) and indicated for each lane. Proteins (10 µg) were resolved by SDS-PAGE and the gels subject to western blot analysis with an anti-SNAP antibody (New England Biolabs) (bottom) and scanned using a fluorimager (top). Black or red arrows point to unlabeled or labeled SNAP, respectively. Labeling with TMR-Star slows down the migration of the SNAP protein. The position of molecular weight markers (in kDa) is shown on the left side of the panels.
(EPS)

Figure S7 Structure of the promoter regions of the *C. difficile sigF* (A), *sigE* (B), *sigG* (C) and *sigK* (D) genes. The upper part of each panel is a representation of the genetic organization of the different genes, the position of the type II intron insertion, and the extent of the DNA fragments cloned in the indicated replicative plasmids for complementation experiments (see also Figure S4A). The bottom part of each panel shows the DNA sequence immediately uptream of the coding region of each gene. The −10 and −35 promoter elements, Spo0A boxes (for the *sigF* gene), and the start codon of each gene, are indicated. Numbering is relative to the position of the start codon.
(EPS)

Figure S8 Structure of the promoter regions of the *C. difficile gpr* (A), *spoIIIAA* (B), *sspA* (C) and *cotE* (D) genes. The upper part of each panel is a representation of the genetic organization of the different genes. The bottom part of each panel shows the DNA sequence of the region immediately upstream of the coding region of each gene, with the −10 and −35 promoter elements indicated. Numbering is relative to the start codon.
(EPS)

Table S1 Sporulation of strain 630Δ*erm* in SM.
(PDF)

Table S2 Strains used in this study.
(PDF)

Table S3 Oligonucleotide primers used in this study.
(PDF)

Table S4 Plasmids used in this study.
(PDF)

Text S1 Supplemental Materials and Methods and Supplemental Results and Discussion.
(PDF)
