# Supplementary material for: The Spore Differentiation Pathway in the Enteric Pathogen Clostridium difficile
Source: PLoS Genet. 2013 Oct 3;9(10):e1003782. doi: 10.1371/journal.pgen.1003782 (PMC3789829; doi:10.1371/journal.pgen.1003782)
Supplement: Table S1 — Sporulation of strain 630Δerm in SM. (PDF) [file pgen.1003782.s009.pdf]

Table S1 - Sporulation of strain 630Δ*erm* in SM.

Time (h)	Heat tests[a]			Microscopy[b]
	Total Cells (CFU/ml)	Heat-resistant Cells (CFU/ml)	% sporulation	% sporulation
0	$8.4x10^{5} ±2.9\ x10^{5}$	$≤10^{1}$	-	-
4	$2.8x10^{6} ±8.4\ x10^{5}$	$≤10^{1}$	-	-
6	$7.3x10^{6} ±2.2\ x10^{6}$	$≤10^{1}$	-	-
8	$8.0x10^{7} ±1.3\ x10^{7}$	$≤10^{1}$	-	-
10	$2.8x10^{8} ±1.6\ x10^{8}$	$≤10^{1}$	-	-
12	$3.2x10^{8} ±2.0\ x10^{8}$	$3.7x10^{2} ±2.0\ x10^{2}$	0.0±0.0	-
16	$9.7x10^{7} ±9.0\ x10^{6}$	$5.7x10^{4} ±9.4\ x10^{3}$	0.1±0.0	0.3±0.1
20	$7.4x10^{7} ±1.1\ x10^{7}$	$2.4x10^{5} ±1.4\ x10^{5}$	0.3±0.2	1.3±0.3
24	$3.3x10^{7} ±2.0\ x10^{7}$	$4.9x10^{5} ±2.0\ x10^{5}$	2.8±1.2	2.1±0.9
36	$2.5x10^{7} ±1.5\ x10^{7}$	$9.5x10^{5} ±6.2\ x10^{4}$	5.5±4.5	2.8±0.7
48	$2.3x10^{7} ±6.2\ x10^{6}$	$2.1x10^{6} ±5.3x10^{5}$	10.3±4.9	3.1±0.9
72	$1.3x10^{7} ±5.4\ x10^{6}$	$4.7x10^{6} ±1.5\ x10^{6}$	43.8±25.6	-

[a] Numbers are from the chart of Figure1A. Values are the average and SD (standard deviation) of three independent experiments. For times 0-10h, no heat resistant spore counts were detected after plating a 100 µl undiluted culture sample.

[b] Percentage of sporulation was calculated by dividing the sum of phase bright and free spores as detected by phase contrast microscopy, by the total number of viable cells scored under the microscope (at least 1000 cells were scored for each time point). Values are the average of three independent experiments.
