# Supplementary material for: The Spore Differentiation Pathway in the Enteric Pathogen Clostridium difficile
Source: PLoS Genet. 2013 Oct 3;9(10):e1003782. doi: 10.1371/journal.pgen.1003782 (PMC3789829; doi:10.1371/journal.pgen.1003782)
Supplement: Table S2 — Strains used in this study. (PDF) [file pgen.1003782.s010.pdf]

Table S2 - Bacterial strains used in this study.

Strain	Relevant Properties	Origin/ Reference
E.coli		
Dh5α		Invitrogen
HB101 (RP4)		Laboratory stock
AHCD018	HB101 (RP4)/ pMTL007::Cdi-*sigE*-453s	This work
AHCD019	HB101 (RP4)/ pMTL007::Cdi-*sigF*-459s	“
AHCD020	HB101 (RP4)/ pMTL007::Cdi-*sigG*-546s	“
AHCD021	HB101 (RP4)/ pMTL007::Cdi-*sigK*-102s	“
AHCD072	HB101 (RP4)/ pFT32	“
AHCD073	HB101 (RP4)/ pFT38	“
AHCD074	HB101 (RP4)/ pFT39	“
AHCD075	HB101 (RP4)/ pFT40	“
AHCD076	HB101 (RP4)/ pMTL84121	“
AHCD077	HB101 (RP4)/ pFT42	“
AHCD095	HB101 (RP4)/ pFT46	“
AHCD108	HB101 (RP4)/ pFT49	“
AHCD109	HB101 (RP4)/ pFT50	“
AHCD110	HB101 (RP4)/ pFT51	“
AHCD112	HB101 (RP4)/ pFT48	“
AHCD114	HB101 (RP4)/ pFT53	“
AHCD115	HB101 (RP4)/ pFT54	"
AHCD116	HB101 (RP4)/ pFT55	"
AHCD131	HB101 (RP4)/ pFT47	“
AHCD142	HB101 (RP4)/ pFT64	“
AHCD149	HB101 (RP4)/ pFT63	“
AHCD150	HB101 (RP4)/ pFT69	“
AHCD151	HB101 (RP4)/ pFT70	"
B. subtilis		
MB24	*trpC2 metC3* /Spo$^+$	Laboratory strain
AH10170	MB24 *tgl-cfp*	“
B. cereus		
ATCC4342	*B. cereus* wild type strain	[93]
C. difficile		
630Δ*erm*	*C. difficile* 630Δ*erm*	[85]
630Δ*erm spo0A*	630Δ*erm spo0A*::intron *ermB*	[61]
AHCD532	630Δ*erm sigE*::intron *ermB*	This work
AHCD533	630Δ*erm sigF*::intron *ermB*	“
AHCD534	630Δ*erm sigG*::intron *ermB*	“
AHCD535	630Δ*erm sigK*::intron *ermB*	“
AHCD543	630Δ*erm* containing pMTL84121	“
AHCD544	AHCD533 containing pMTL84121	“
AHCD545	AHCD532 containing pMTL84121	“
AHCD546	AHCD534 containing pMTL84121	“
AHCD547	AHCD535 containing pMTL84121	“
AHCD548	AHCD533 containing pFT32	“
AHCD549	AHCD532 containing pFT39	“
AHCD550	AHCD534 containing pFT40	“
AHCD551	AHCD535 containing pFT38	“
AHCD577	AHCD535 containing pFT42	“
AHCD586	630Δ*erm* containing pFT46	“
AHCD597	630Δ*erm* containing pFT49	“

AHCD598	630Δ*erm* containing pFT48	“
AHCD600	630Δ*erm* containing pFT50	“
AHCD601	630Δ*erm* containing pFT51	“
AHCD602	630Δ*erm* containing pFT53	“
AHCD603	630Δ*erm* containing pFT54	“
AHCD604	630Δ*erm* containing pFT55	“
AHCD610	AHCD533 containing pFT53	“
AHCD611	AHCD533 containing pFT54	“
AHCD612	AHCD533 containing pFT55	“
AHCD613	AHCD532 containing pFT53	“
AHCD614	AHCD532 containing pFT54	“
AHCD615	AHCD532 containing pFT55	“
AHCD619	AHCD534 containing pFT55	“
AHCD621	AHCD535 containing pFT55	“
AHCD630	AHCD533 containing pFT48	“
AHCD631	AHCD533 containing pFT49	“
AHCD632	AHCD533 containing pFT50	“
AHCD633	AHCD533 containing pFT51	“
AHCD634	AHCD532 containing pFT48	“
AHCD635	AHCD532 containing pFT49	“
AHCD636	AHCD532 containing pFT50	“
AHCD637	AHCD532 containing pFT51	“
AHCD640	AHCD534 containing pFT50	“
AHCD641	AHCD534 containing pFT51	“
AHCD644	AHCD535 containing pFT50	“
AHCD645	AHCD535 containing pFT51	“
AHCD646	630Δ*erm* containing pFT47	“
AHCD656	630Δ*erm* containing pFT64	“
AHCD674	AHCD534 containing pFT64	“
AHCD675	AHCD535 containing pFT64	“
AHCD678	630Δ*erm* containing pFT63	“
AHCD679	AHCD534 containing pFT63	“
AHCD680	AHCD535 containing pFT63	“
AHCD683	AHCD534 containing pFT53	“
AHCD684	AHCD535 containing pFT53	“
AHCD685	AHCD534 containing pFT54	“
AHCD686	AHCD535 containing pFT54	“
AHCD692	630Δ*erm spo0A* containing pFT48	“
AHCD693	630Δ*erm spo0A* containing pFT49	“
AHCD695	630Δ*erm* containing pFT69	“
AHCD696	AHCD533 containing pFT69	“
AHCD697	AHCD532 containing pFT69	“
AHCD698	AHCD534 containing pFT69	“
AHCD695	AHCD535 containing pFT69	“
