# Supplementary material for: The Spore Differentiation Pathway in the Enteric Pathogen Clostridium difficile
Source: PLoS Genet. 2013 Oct 3;9(10):e1003782. doi: 10.1371/journal.pgen.1003782 (PMC3789829; doi:10.1371/journal.pgen.1003782)
Supplement: Table S3 — Oligonucleotide primers used in this study. (PDF) [file pgen.1003782.s011.pdf]

Table S3 – Oligonucleotides used in this study.

Primer	Sequence (5´to 3´)[a]	Description
CT001	AAAAAAGCTTATAATTATCCTTAGGAAACGAACTTGTG CGCCCAGATAGGGTG	IBS-sigF-459s
CT002	CAGATTGTACAAATGTGGTGATAACAGATAAGTCGAA CTTTTTAACTTACCTTTCTTTGT	EBS1d-sigF-459s
CT003	TGAACGCAAGTTTCTAATTTCGATTTTTCCTCGATAGA GGAAAGTGTCT	EBS2-sigF-459s
CT004	AAAAAAGCTTATAATTATCCTTAGATTTCGTAATGGTG CGCCCAGATAGGGTG	IBS-sigE-453s
CT005	CAGATTGTACAAATGTGGTGATAACAGATAAGTCGTA ATGGCTAACTTACCTTTCTTTGT	EBS1d-sigE-453s
CT006	TGAACGCAAGTTTCTAATTTCGATTAAATCTCGATAGA GGAAAGTGTCT	EBS2-sigE-453s
CT007	AAAAAAGCTTATAATTATCCTTAGATGCCATATTCGTG CGCCCAGATAGGGTG	IBS-sigG-546s
CT008	CAGATTGTACAAATGTGGTGATAACAGATAAGTCATAT TCGTTAACTTACCTTTCTTTGT	EBS1d-sigG-546s
CT009	TGAACGCAAGTTTCTAATTTCGATTGCATCTCGATAGA GGAAAGTGTCT	EBS2-sigG-546s
CT010	AAAAAAGCTTATAATTATCCTTAGCAAACGATACAGTG CGCCCAGATAGGGTG	IBS-sigK-102s
CT011	CAGATTGTACAAATGTGGTGATAACAGATAAGTCGAT ACATTTAACTTACCTTTCTTTGT	EBS1d-sigK-102s
CT012	TGAACGCAAGTTTCTAATTTCGATTTTTGCTCGATAGA GGAAAGTGTCT	EBS2-sigF-102s
EBS	CGAAATTAGAAACTTGCGTTCAGTAAAC	PCR ClosTron
ErmRAM-F	ACGCGTTATATTGATAAAAATAATAATAGTGGG	PCR *erm* intron
ErmRAM-R	ACGCGTGCGACTCATAGAATTATTTCCTCCCG	PCR *erm* intron
pMTL007-F	TTAAGGAGGTGTATTTCATATGACCATGATTACG	Sequencing pMTL007
pMTL007-R	AGGGTATCCCCAGTTAGTGTTAAGTCTTGG	Sequencing pMTL007
M13R	CAG GAA ACA GCT ATG AC	Sequencing pMTL84121
M13F	GTTTTCCCAGTCACGAC	Sequencing pMTL84121
OBD522	ATCTGTAGGAGAACCTATGGGAAC	Southern blot probe
OBD523	CACGTAATAAATATCTGGACGTAAAA	Southern blot probe
CDsigF_257 Fw	CTACTTATGCAGTACCAATG	*sigF* 5'-intron
CDsigF_616 Rev	GACGCTCTCTCTTATCCAG	*sigF* 3'-intron
CDsigE_331 Fw	GCAACTTATGCTTCGAGATG	*sigE* 5'-intron
CDsigE_625 Rev	CCAAGCATTCCAGCAACTTC	*sigE* 3'-intron
CDsigG_99 Fw	GGTGATGAAGAAGCCAGAC	*sigG* 5'-intron
CDsigG_700 Rev	CATCAGCAACTTCTATCTGAG	*sigG* 3'-intron
CDsigK_35 Fw	CCCCTGAAGAAGAGATTGAG	*sigK* 5'-intron
CDsigK_238 Rev	GCATATGTTGCTAATCGAGTT	*sigK* 3'-intron
PCDsigF Fw	GCTGCCGCCGCCGAGCTTTATTCAGA	5'-promoter *sigF*-NotI
PCDsigF Rev	GGCAACAGTTACTTCCATCAAAAATCCCTCCTT	3'-promoter *sigF*
CDsigF Fw	AAGGAGGGATTTTTGATGGAAGTAACTGTTGCC	5'-*sigF*
CDsigF_EcoRI Rev	GCTGAATTCTCACGATATATATTC	3'-*sigF*-EcoRI
PCDsigE Fw	GCTGCGGCCGCGACCATTGACAGTTGGAC	5'-promoter *sigE*-NotI
PDsigE Rev	TTCTTTCAATCGTAACATGTATCCCCCCTTAAAACC	3'-promoter *sigE*
CDsigE Fw	GGTTTTAAGGGGGGATACATGTTACGATTGAAAGAA	5'-*sigE*
CDsigE_EcoRI Rev	GCTGAATTCTTATACAAATTTTTTCAT	3'-*sigE*-EcoRI
PCDsigG Fw	GCTGCGGCCGCCGAGATGTATAGAAAATG	5'-promoter *sigG*-NotI
CDsigG_EcoRI Rev	GCTGAATTCTTATACATATTTTCTCAT	3'-*sigG*-EcoRI
PCDsigK5' Fw	GCTGCGGCCGCTGACTGATACTTTTG	5'-promoter *sigK*-NotI
CDsigK5' Rev	CACTTTTCTTAAATCACTAGCTATAGAAATTGATGA	3'-promoter *sigK*
CDsigK3' Fw	TCATCAATTTCTATAGCTAGTGATTTAAGAAAAGTG	5'-*sigK*
CDsigK3'_EcoRI Rev	GCTGAATTCTTAACTTTCTTGAACAAG	3'-*sigK*-EcoRI
SNAPtag_SacI Fw	GCTCGAGAGCTCGGAGGAACTACTATGG	5'-*SNAPCd*-SacI
SNAPtag_BamHI Rev	GCTGTTGGATCCAAGCTTTCCTTACCC	3'-*SNAPCd*-BamHI
PCDsigF-XhoI Rev	GGAATAATTCTCGAGCAAAAATCCCTCCTT	3'-promoter *sigF*-XhoI
PCDsigE-XhoI Rev	CAATATACTCGAGGTATCCCCCCTTAAAACC	3'-promoter *sigE*-XhoI
PCDsigG-XhoI Rev	ATTAACCTCGAGATATTTTCAGTCCTCTCT	3'-promoter *sigG*-XhoI

PCDsigK-XhoI Rev	AAGAGCCTCGAGTAGGTCACCCCCTTTTCA	3’-promoter *sigK*-XhoI
Pgpr-EcoRI Fw	AAGAGCTATGAATTCGGGTATTTATCACTT	5’-promoter *gpr*-EcoRI
Pgpr-XhoI Rev	CATATAAACCTCGAGTATAAATTAAATGCT	3’-promoter *gpr*-XhoI
PspoIIIAA-EcoRI Fw	TAGATGGTGGAATTCCTAGGGCTTACCAAA	5’-promoter *spoIIIAA*-EcoRI
PspoIIIAA-XhoI Rev	GTTTATTCATCTCTTGCTCGAGTCCTTTG	3’-promoter *spoIIIAA*-XhoI
PsspA-EcoRI Fw	AGATGAGGAGAATTCGGATAAAAGAGTTCA	5’-promoter *sspA*-EcoRI
PsspA-XhoI Rev	CTTCCTTCTCTCGAGTTTATTTTGTGTTGC	3’-promoter *sspA*-XhoI
SNAP-linker BamHIFw	GGTGGTGGTGGATCCGCAGCTGCTGATAAAGATTGT GAAATGAAG	5’- *SNAPCd* -linker BamHI
PcotB-EcoRI Fw	TACCTAGAATTCTAAGTGCAGCTATTAG	5’-promoter *cotB*-EcoRI
CotB Linker Rev	GCTGCGGATCCACCACCACCAAGCATGTTTTTATAAC TCTCC	3’-*cotB*-linker BamHI
PcotE-EcoRI Fw	CATAACTGAATTCATTAACACTAATGC	5’-promoter *cotE*-EcoRI
CotE Linker Rev	GCTGCGGATCCACCACCACCAAGGAATTGCCCATAAA TACCTTC	3’-*cotE*-linker BamHI
PcotE-XhoI Rev	CATGTAAATCACCTCGAGATAAATTATCATTT	3’-promoter *cotE*-XhoI
DNApolIII-F	TCCATCTATTGCAGGGTGGT	qRT-PCR, *dnaF* (CD1305)
DNApolIII-R	CCCAACTCTTCGCTAAGCAC	“
IMV552	CCAGGAGCAAAAGAGGCTTTA	qRT-PCR, *sspA* (CD2688)
IMV553	TCCAGCCATTTGTTGTTCAG	“
LS100	CACCACCTCAATGTGGAAAA	qRT-PCR, *spoIIIAA* (CD1192)
LS101	GCTCCTGCTATCTCATTACGC	“
LS114	TGCATTGGCTTCAAGAAAAA	qRT-PCR, *gpr* (CD2470)
LS115	GCAATAACATCCACGCCTAAA	“
LS143	CAGGCCCAAATGGTAGAAAA	qRT-PCR, *cotE* (CD1433)
LS144	GAAGGCATTCCAGCATTCTC	“

[a] Restriction sites are underlined.
