# Supplementary material for: The Spore Differentiation Pathway in the Enteric Pathogen Clostridium difficile
Source: PLoS Genet. 2013 Oct 3;9(10):e1003782. doi: 10.1371/journal.pgen.1003782 (PMC3789829; doi:10.1371/journal.pgen.1003782)
Supplement: Table S4 — Plasmids used in this study. (PDF) [file pgen.1003782.s012.pdf]

## Table S4 – Plasmids used in this study.

| Plasmid | Relevant features | Origin/ reference |
|---|---|---|
| pMTL007 | ClosTron plasmid containing *catP* and intron containing *ermB* RAM ($Cm^R/Tm^R$)[1] | [44] |
| pMTL84121 | *Clostridium* modular plasmid containing *catP* ($Cm^R/Tm^R$) | [45] |
| pRPF185 | pMTL960 plasmid carrying $P_{tet}$-*gusA* ($Cm^R/Tm^R$) | [59] |
| pMTL007::Cdi-*sigE*-453s | pMTL007 retargeted to the *sigE* gene ($Cm^R/Tm^R$) | This work |
| pMTL007::Cdi-*sigF*-459s | pMTL007 retargeted to the *sigF* gene ($Cm^R/Tm^R$) | “ |
| pMTL007::Cdi-*sigG*-546s | pMTL007 retargeted to the *sigG* gene ($Cm^R/Tm^R$) | “ |
| pMTL007::Cdi-*sigK*-102s | pMTL007 retargeted to the *sigK* gene ($Cm^R/Tm^R$) | “ |
| pFT32 | pMTL84121-*sigF* ($Cm^R/Tm^R$) | This work |
| pFT38 | pMTL84121-$sigK^{skin+}$ ($Cm^R/Tm^R$) | “ |
| pFT39 | pMTL84121-*sigE* ($Cm^R/Tm^R$) | “ |
| pFT40 | pMTL84121-*sigG* ($Cm^R/Tm^R$) | “ |
| pFT42 | pMTL84121-$sigK^{skin-}$ ($Cm^R/Tm^R$) | “ |
| pFT46 | $P_{tet}$-$SNAP^{Cd}$ ($Cm^R/Tm^R$) | |
| pFT47 | pMTL84121-$SNAP^{Cd}$ ($Cm^R/Tm^R$) | “ |
| pFT48 | pFT47 containing the *sigF* promoter region | “ |
| pFT49 | pFT47 containing the *sigE* promoter region | “ |
| pFT50 | pFT47 containing the *sigG* promoter region | “ |
| pFT51 | pFT47 containing the *sigK* promoter region | “ |
| pFT53 | pFT47 containing the the *gpr* promoter region | “ |
| pFT54 | pFT47 containing the *spoIIIAA* promoter region | “ |
| pFT55 | pFT47 containing the *sspA* promoter region | “ |
| pFT58 | pMTL84121-linker-$SNAP^{Cd}$ (for translational fusions) ($Cm^R/Tm^R$) | “ |
| pFT63 | pFT58 containing promoter and coding sequence of *cotB* | “ |
| pFT64 | pFT58 containing promoter and coding sequence of *cotE* | “ |
| pFT69 | pFT47 containing *cotE* promoter region | “ |
