# Supplementary material for: The Spore Differentiation Pathway in the Enteric Pathogen Clostridium difficile
Source: PLoS Genet. 2013 Oct 3;9(10):e1003782. doi: 10.1371/journal.pgen.1003782 (PMC3789829; doi:10.1371/journal.pgen.1003782)
Supplement: Text S1 — Supplemental Materials and Methods and Supplemental Results and Discussion. (PDF) [file pgen.1003782.s013.pdf]

The spore differentiation pathway in the enteric pathogen *Clostridium difficile*

Fátima C. Pereira, Laure Saujet, Ana R. Tomé, Mónica Serrano, Marc Monot, Evelyne Couture-Tosi, Isabelle Martin-Verstraete, Bruno Dupuy, and Adriano O. Henriques

Supplemental Materials and Methods

Construction of gene knockout mutants in *C. difficile*

The ClosTron gene knockout system [44] was used to inactivate the *sigF*, *sigE*, *sigG* and *sigK* genes. Primers to retarget the group II intron of pMTL007 to these genes (all primers used in this work are listed in Table S3) were designed with the Targetron design software (Sigma-Aldrich). The PCR primer sets were used with the EBS universal primer and intron template DNA to generate by overlap extension PCR a 353-bp product for each gene that allows intron retargeting. These PCR products were cloned between the HindIII and BsrGI restriction sites of pMTL007 giving plasmids pMTL007::Cdi-sigF-459s, pMTL007::Cdi-sigE-453s, pMTL007::Cdi-sigG-546s and pMTL007::Cdi-sigK-102s (all plasmids are listed in Table S4). DNA sequencing was performed to verify plasmid constructs, using the pMTL007-specific primers pMTL007-F and pMTL007-R. The derivative pMTL007 plasmids were transformed into *E. coli* HB101 (RP4) and subsequently mated with *C. difficile* 630Δ*erm* [44]. *C. difficile* transconjugants were selected by subculturing on BHI agar containing thiamphenicol (15 μg/ml) and cefoxitin (25 μg/ml) and then plated on BHI agar containing erythromycin (5.0 μg/ml). This produced the 630Δ*erm* derivatives AHCD533 (*sigF*::*erm*), AHCD532 (*sigE*::*erm*), AHCD534 (*sigG*::*erm*) and AHCD535

(*sigK*::*erm*), respectively (Table S2). Chromosomal DNA was isolated from the transconjugants, and the structure of the various mutants in the vicinity of the disrupted gene was verified by PCR.

Complementation in *C. difficile*

To complement the *sigF* mutation, the coding sequence of the *sigF* gene (765 bp length) and its expected promoter region comprising 514 bp upstream of *spoIIAA* were independently amplified by PCR using primer pairs Prom_CDsigF Fw/Prom_CD sigF Rev and CDsigF Fw/CDsigF-EcoRI Rev (Table S3). The two pieces were joined into a 1279 bp fragment by splicing by overlaping extension (SOE) PCR and cloned between the NotI and EcoRI sites of pMTL84121 [45], to produce pFT32 (Table S4). To complement the *sigE* mutation, the entire coding sequence of *sigE* gene (742 bp length) and its expected promoter region comprising the 459 bp upstream of *spoIIGA* were independently PCR amplified using primer pairs Prom_CDsigE Fw/Prom_CD sigE Rev and CDsigE Fw/CDsigE-EcoRI Rev, joined by SOE PCR and the resulting 1143 bp fragment cloned between the NotI and EcoRI sites of pMTL84121 to create pFT39. To complement the *sigG* mutation, a fragment encompassing the coding sequence of *sigG* (774 bp length) and 459 bp of its regulatory region was PCR amplified using primer pair Prom_CDsigG Fw/CDsigG-EcoRI Rev. The resulting 1233 bp fragment was cloned between the NotI and EcoRI sites of pMTL84121 to yield pFT40. Finally, to complement the *sigK* mutation two constructs were generated. First, the interrupted 5´ coding sequence of *sigK* gene (404 bp after the translational start site) and its expected promoter region comprising the 415 bp upstream of *sigK* gene was PCR amplified using primers Prom_CDsigK Fw and CDsigK5’ Rev yielding a 819 bp fragment. In a second PCR,

the region containing the interrupted 3´ coding sequence of *sigK*, the gene (CD1231) coding for the recombinase, and a 466 bp fragment upstream of this gene was PCR amplified using primer pair CDsigK3’ Fw/CDsigK3’_EcoRI Rev. This fragment, of 2285 bp was joined to the 819 bp fragment by SOE PCR and cloned between the NotI and EcoRI sites of pMTL84121 to produce pFT38. A second construct was generated in which an intact *sigK* gene with 419 bp of its regulatory region was amplified using primer pair Prom_CDsigK Fw/ CDsigK3’_EcoRI Rev from DNA prepared from 48h cultures in BHI, a time at which most of the cells already bear a re-arranged sigK gene. The resulting 1061 bp fragment was cloned between the NotI/EcoRI sites of pMTL84121 yielding pFT42. Using the *E. coli* HB101 (RP4) strain as donor, pFT32, pFT38, pFT39, pFT40 and pFT42 were transferred by conjugation into the various *sig*::*erm* mutants of *C. difficile* 630Δ*erm*, giving strains AHCD548, AHCD551, AHCD549, AHCD550 and AHCD577 (Table S2).

Southern Blot

For Southern blot analysis, 6 μg of genomic DNA from *C. difficile* strain 630Δ*erm* and the congenic *sigF*, *sigE*, *sigG* or *sigK* mutant strains were digested to completion with HindIII. The Southern blot probe was generated by PCR using pMTL007 plasmid as a template and primer pair OBD522 and OBD523 (Table S3), yielding a 374 bp PCR product that hybridizes within the group II intron. Southern blot analyses were performed using Amersham ECL Direct Nucleic Acid labeling and detection reagents, according to the manufacturer's guidelines. The hybridization signal was detected using Super Signal West Femto Maximum Sensitivity Substrate (Thermo Scientific).

Spore production, purification and decoating

For spore production, 5 ml of BHI media was inoculated with an isolated colony of *C. difficile* 630Δ*erm* and cultured overnight at 37ºC in anaerobic conditions. 100 ml of fresh BHI media was then inoculated with 1 ml of the overnight culture and incubated at 37ºC under anaerobic conditions for 10 days. Cells were collected by centrifugation at 4800xg, ressuspended in cold water and stored over night at 4ºC. Spores were then purified with a 40-50% Gastrografin (Schering) step gradient, as previously described [84]. The pellet was ressuspended in cold water, washed 10 times with cold water, and stored at 4ºC until further use.

To remove the spore coat, *C. difficile* purified spores were resuspended in 50 μl of extraction buffer (0.1 M NaOH, 0.1 M NaCl, 1% SDS, 0.1 M DTT) to a final OD_{600nm} of 4.0. Spores were then incubated at 70ºC for 30 minutes, extensively washed with water, to remove all traces of NaOH and SDS, and then stained with the FM4-64 dye as described.

Transcriptional *SNAP*^{Cd} fusions

We first obtained a synthetic version of the *SNAP26b* gene (encoding a mutant form of the human gene for O6-alkylguanine-DNA-alkyltransferase (New England Biolabs); [86]) codon usage-optimized for expression in *C. difficile* (DNA 2.0, Menlo Park, CA). The synthetic gene cassette, hereinafter termed *SNAP*^{Cd}, includes a ribosome-binding site (RBS) and flanking XhoI and HindIII sites. To construct a P_{tet}-*SNAP*^{Cd} fusion (*SNAP*^{Cd} under the control of P_{tet} inducible promotor), the synthetic *SNAP*^{Cd} cassette was PCR-amplified using primer pair SNAPtag_SacI Fw/SNAPtag_BamHI Rev and inserted between the SacI and BamHI sites of

pRPF185 [59], replacing the *gusA* gene. Anhydrotetracycline (ATc; 250 ng/ml) was used for induction of the P_{tet} promoter present in *C. difficile* strains bearing pFT46 (Figure S5A). The synthetic *SNAP*^{Cd} sequence was also cloned between the XhoI/HindIII sites of pMTL84121, to produce pFT47 (Figure S5B). To construct *sigF*-, *sigE*-, *sigG*- and *sigK*- transcriptional *SNAP*^{Cd} fusions, the promoter regions of these genes were PCR-amplified using genomic DNA from strain 630Δ*erm* and primer pairs PCDsigF-Fw and PCDsigF-XhoI Rev, PCDsigE-Fw and PCDsigE-XhoI Rev, PCDsigG-Fw and PCDsigG-XhoI Rev and PCDsigK5´-Fw and PCDsigK-XhoI Rev, to produce 539, 482, 494 and 437 bp products, respectively (Figure S7). These were inserted between the EcoRI and XhoI sites of pFT47 to create pFT48, pFT49, pFT50 and pFT51 (Table S4). To monitor the activities of σ^F, σ^E, σ^G and σ^K, the promoter regions for the *gpr*, *spoIIIAA*, *sspA* and *cotE* genes were PCR-amplified from 630Δ*erm* genomic DNA using primer pairs Pgpr-EcoRI Fw and Pgpr-XhoI Rev, PspoIIIAA-EcoRI Fw and PspoIIIAA-XhoI Rev, PsspA-EcoRI Fw and PsspA-XhoI Rev, and PcotE-EcoRI Fw and PcotE-XhoI Rev. The resulting 433, 533, 489, and 303 bp fragments, respectively, were inserted between the EcoRI and XhoI sites of pFT47 to create pFT53, pF54, pFT55, and pFT69 (Table S4 and Figure S8). The absence of unwanted mutations was verified by sequencing the insert in all the plasmids. All plasmids bearing promoter-*SNAP*^{Cd} fusions were introduced into *E. coli* HB101 (RP4) and then transferred to *C. difficile* 630Δ*erm* and *sigF* (AHCD533), *sigE* (AHCD532), *sigG* (AHCD534) and *sigK* (AHCD535) mutant strains by conjugation [44] (Table S2).

Translational *SNAP*^{Cd} fusions

To create C-terminal SNAP-tag protein fusions, the *SNAP*^{Cd} sequence was amplified from pFT47 using primers SNAP-linker BamHI Fw and SNAP-tag Rev and cloned between BamHI and HindIII sites of pMTL84121. The resulting plasmid, pFT58, contains the *SNAP*^{Cd} sequence without the start codon (Figure S5C). The promoter and coding regions of *cotB* (CD1511) and *cotE* (CD1433) were amplified from 630Δ*erm* genomic DNA using primer pairs PcotB-EcoRI Fw and cotB-Linker-Rev, and PcotE-EcoRI Fw and cotE-Linker-Rev (Table S3). The resulting PCR products were inserted between the EcoRI and BamHI sites of pFT58, yielding pFT63 and pFT64, respectively (Table S4). In the final constructs, the *cotB* and *cotE* sequences are separated from the SNAP-coding sequence by a sequence coding for a 9 amino acid linker (LGGGGSAAA). Plasmids pFT63 and pFT64 were then transferred to *C. difficile* 630Δ*erm*, *sigG* (AHCD534) and *sigK* (AHCD535) mutant strains by conjugation [44] (Table S2).

Supplemental Results and Discussion

Spore staining by FM4-64

A characteristic of sporulation in *C. difficile* 630Δ*erm* that distinguishes it from the *B. subtilis* model is the strong staining of the developing spore with the FM4-64 dye following engulfment completion (Figure S2A). Staining of the engulfed forespore with FM4-64 was unexpected, because the lipophilic dye does not label engulfed forespores of *B. subtilis* [43].

Because *C. difficile* cells emit strong green fluorescence under long wavelength UV light [88], it seemed possible that spore staining with FM4-64 could be at least in part an artifact caused by emission in the red channel. To investigate this, we mixed sporulating cells of *B. subtilis* (which are not auto-fluorescent) and *C. difficile* and labeled the mixed suspension with FM4-64, prior to fluorescence microscopy examination in both the green and red channels. Under our experimental conditions, sporulating cells of *C. difficile*, but not the developing spore, emit strong green fluorescence, but this signal is not detected in the red channel (Figure S2A, top). Conversely, the FM4-64 signal of membranes and spores does not contribute to the green fluorescence emission (Figure S2A, top). Additionally, engulfed spores of *B. subtilis*, observed in the same microscope field, do not stain with FM4-64 (Figure S2A, top) [43]. These observations confirm that the developing spore of *C. difficile* stains strongly with FM4-64, even following engulfment completion. One possibility is that the membrane of the engulfed spore is in contact with the mother cell membrane. Fluorescence microscopy images of sporulating cells of *Metabacterium polyspora* and of *Epulopiscium* spp., both of which form multiple spores inside the same mother cell, suggest that at least some

of the forespores are in contact with the cell membrane, and that they also contact each other [3], [4]. In *M. polyspora*, the engulfed forespores stain with FM4-64 [3]. However, affinity of the FM dyes to the spore coats has also been reported [3] and thus, it is unclear whether forespore staining is due to contact of the spore membrane or another spore structure that is already present at this stage (just after engulfment completion) with the mother cell membrane.

Strong staining of the spore with FM4-64 is maintained at later stages of sporulation, when the spore becomes phase bright and free spores also stain strongly (Figure S2A). To confirm staining of mature spores with FM4-64, we mixed *B. subtilis* and *C. difficile* spores, following which the suspension was stained with FM4-64. The *B. subtilis* spores expressed a fusion of a spore surface protein (Tgl) to CFP, to allow their identification by fluorescence microscopy, in the same microscope field. The *C. difficile* spores which, contrary to cells are not auto-fluorescent (see above), exhibited intense red fluorescence but no blue fluorescence, while those of *B. subtilis* remained unstained by the FM4-64 dye (Figure S2A, bottom). Thus, in *C. difficile,* both engulfed forespores as well as mature, free spores are selectively stained by FM4-64.

Because of the reported affinity of FM dyes to the spore coats [3], we consider the possibility that staining reflected the structure and composition of the spore surface layers. For example, recent work as reported on the presence of an exosporium surrounding the spore coats of *C. difficile* [55], [92] (see also below). However, if an exosporium contributes to staining of the *C. difficile* spores by the FM4-64 dye, then its properties must differ considerably from those of the exosporial layers of an organism like *B. cereus*, whose spore do not stain with the dye (Figure S2B). A decoating regime applied to *C. difficile* spores, during which the spore remains phase bright, strongly reduces spore staining by FM4-64 (Figure S2C).

Although this regime is expected to remove both the exosporium and coat layers, the experiment suggests that staining of *C. difficile* spores with FM4-64 is due, at least in part, to the structure and composition of the spore surface layers.

We suspect that staining of the engulfed forespores by the FM4-64 dye may also be related to the presence of a surface structure, already in place (at least in part) following engulfment completion. In *B. subtilis*, *B. cereus* and *B. anthracis*, and although not necessarily detected by transmission electron microscopy (TEM) prior to the complete engulfment of the forespore with the concomitant activation of σ^K, assembly of the spore coats begins early in the mother cell line of gene expression, under the control of σ^E [15], [16].

Spore ultrastructure

Sporulating cells and spores of *C. difficile* were also examined using TEM. A feature noteworthy is that in some of the sporulating cells, a membrane-looking structure surrounding the forespore was noticed (Figure S3A and B). This membrane-looking structure appears to contact the cell membrane (Figure S3B). We were unable to determine when this structure was formed during sporulation, as it was only detected in cells, as those represented in Figure S3, at late stages in development, as judged by the presence of spores with discernible cortex and coat layers, and by the almost complete dissolution of the mother cell cytoplasm. We do not know whether the presence of this structure is related to the FM4-64 staining of engulfed forespores.

In most of the free spores the cortex was surrounded by a lamellar inner coat layer formed by 5-6 lamellae, covered by an electrodense outer layer (Figure S3C and D). In some of the free spores, an extra layer with a loose shape surrounded the

spore, at a distance from the outer coat surface (Figure S3C). This layer appears as an exosporium, formed by a thin basal layer, with more electrondense material closely apposed to its external surface. The basal layer appears similar to the membrane-like structure seen in sporulating cells (above). It is tempting to speculate that the membrane-like structure seen in sporulating cells represents an intermediate in the assembly of the exosporium. The exosporium-like layer was only seen by TEM in about 10% of the free spores, suggesting that this structure is labile and does not survive sample collection and processing for electron microscopy. In two recent publications, the exosporium surrounding *C. difficile* spores has a thick electrondense appearance [55], [92] and thus differs from the structure herein described. It is possible that morphogenesis of the exosporial layer differs from strain to strain or with culturing conditions.

The SNAP system as a reporter for single cell studies of gene expression

In *B. subtilis*, studies of compartmentalized gene expression during sporulation have relied mainly on the use of transcriptional or translational fusions to the *gfp* gene, or its variants (e.g., [51]). Formation of the GFP chromophore however, involves an oxidation reaction [52], limiting the use of GFP in anaerobic bacteria. As a fluorescence transcriptional reporter for single cell analysis in *C. difficile* was missing, we turned to the SNAP-tag [86]. The SNAP-tag reacts with benzyl purine or pyrimidine substrates that can be coupled to different fluorescent molecules. Previous studies have shown its usefulness for studies of gene expression in different organisms, including anaerobic bacteria [53], [54]. We synthesized a variant of the *SNAP26b* gene, which we termed $SNAP^{Cd}$, preceded by

a ribosome-binding site that was codon-usage optimized for expression in *C. difficile*. This cassette was placed under the control of the anhydrotetracycline (ATc) responsive P_{tet} promoter in a replicative plasmid [59], to yield pFT46 (Figure S5A). Plasmid pFT46 was introduced into the wild type strain 630Δ*erm*. The resulting strain was grown in BHI until mid-exponential phase, induced by addition of 250 ng/ml of ATc, which in preliminary experiments we found to maximally induce, under our experimental conditions, the P_{tet} promoter (see also [59]). Following induction, samples were collected, the cells labeled with increasing concentrations of the TMR-Star substrate for 30 min, and examined by phase contrast and fluorescence microscopy. Cells to which TMR-Star was not added, ("0" in Figure S6A), or cells that were not induced with ATc but labeled with the highest concentration tested of the substrate (250 nM), did not display fluorescence, as judged by microscopy and quantification of the signal across the population (Figure S6A). Therefore, even in the presence of the TMR-Star substrate, no background fluorescence was registered. Moreover, staining of non-induced cells with 250 nM of TMR-Star did not result in any detectable fluorescence signal, regardless of the incubation time (up to 1 hour; not shown). The average fluorescence signal for the cell population increased linearly with the concentration of TMR-Star in the labeling reaction (Figure S6A and B). For any concentration of TMR-Star tested, about 20% of the cell population did not display fluorescence above the background value ("0" time labeling) (Figure S6C). This number remained constant regardless of the concentration of ATc or the duration of labeling with TMR-Star (not show). We presume that the number of unlabeled cells reflects a normal response of the P_{tet} promoter across the cell population (with transcription not activated in the 20% sub-population), rather than for instance a sub-population of cells refractory to TMR internalization. We base this assumption on the observation that no unlabeled SNAP

could be detected by immunoblotting, for the highest concentration of substrate used (see below). To monitor the extent of labeling of the SNAP protein, we prepared whole cell extracts from cells of stain 630Δ*erm* bearing the P_{tet}-*SNAP*^{Cd} fusion, following labeling with increasing concentrations of TMR-Star. Proteins in extracts were resolved by SDS-PAGE, the gels scanned on a fluorimager and then processed for immunoblot analysis with an anti-SNAP antibody. No SNAP production was detected, by immunobloting, for the uninduced strain (Figure S6D). For the sample corresponding to the induced culture, but unlabeled cells, the immunoblot analysis showed accumulation of the SNAP protein, which migrates just below the 20kDa size marker (Figure S6D). The expected size for the SNAP is of 20 kDa [86]. This species was non-fluorescent, as expected and revealed by the fluorimager scan (Figure S6D, black arrow). Labeling with 50 nM of TMR-Star resulted in the appearance of a band with a slightly higher size, consistent with formation the covalent modification of the SNAP by the TMR-Star substrate (this reaction involves the formation of a stable thioether bond between the reactive cysteine of the SNAP protein and the substrate) [86]. This band was fluorescent, confirming reaction with the SNAP (Figure S6D, red arrows). However, the unlabeled SNAP (with higher mobility relative to the labeled species in the immunoblot, and non-fluorescent) was still detected (Figure S6D, black arrow). Complete labeling of the SNAP-tag produced from the P_{tet}-*SNAP*^{Cd} fusion was only achieved for a concentration of TMR-Star of 250 nM (Figure S6D). In all, these experiments show the usefulness of the SNAP tag as a reporter for gene expression, and the need to optimize the concentration of the TMR-Star substrate to a particular reporter construct, so that complete labeling can be achieved.

References

92. Lawley TD, Croucher NJ, Yu L, Clare S, Sebaihia M, et al. (2009) Proteomic and genomic characterization of highly infectious Clostridium difficile 630 spores. J Bacteriol 191: 5377-5386.
93. Schuch R, Nelson D, Fischetti VA (2002) A bacteriolytic agent that detects and kills Bacillus anthracis. Nature 418: 884-889.
